# Clinical benefit and safety associated with mRNA vaccines for advanced solid tumors: A meta‐analysis

**DOI:** 10.1002/mco2.286

**Published:** 2023-07-18

**Authors:** Tian‐yi Zhang, Hang Xu, Xiao‐nan Zheng, Xing‐yu Xiong, Shi‐yu Zhang, Xian‐yanling Yi, Jin Li, Qiang Wei, Jian‐zhong Ai

**Affiliations:** ^1^ Department of Urology, West China Hospital Sichuan University Chengdu China; ^2^ Institute of Urology, West China Hospital Sichuan University Chengdu China

**Keywords:** cancer treatment, mRNA vaccines, objective response rate, safety, solid tumor

## Abstract

Tumor mRNA vaccines have been developed for over 20 years. Whether mRNA vaccines could promote a clinical benefit to advanced cancer patients is highly unknown. PubMed and Embase were retrieved from January 1, 2000 to January 4, 2023. Random effects models were employed. Clinical benefit (objective response rate [ORR], disease control rate [DCR], 1‐year/2‐year progression‐free survival [PFS], and overall survival [OS]) and safety (vaccine‐related grade 3–5 adverse events [AEs]) were evaluated. Overall, 984 patients (32 trials) were enrolled. The most typical cancer types were melanoma (13 trials), non‐small cell lung cancer (5 trials), renal cell carcinoma (4 trials), and prostate adenocarcinoma (4 trials). The pooled ORR and DCR estimates were 10.0% (95%CI, 4.6–17.0%) and 34.6% (95%CI, 24.1–45.9%). The estimates for 1‐year and 2‐year PFS were 38.4% (95%CI, 24.8−53.0%) and 20.0% (95%CI, 10.4–31.7%), respectively. The estimates for 1‐year and 2‐year OS were 75.3% (95%CI, 62.4–86.3%) and 45.5% (95%CI, 34.0–57.2%), respectively. The estimate for vaccine‐related grade 3–5 AEs was 1.0% (95%CI, 0.2–2.4%). Conclusively, mRNA vaccines seem to demonstrate modest clinical response rates, with acceptable survival rates and rare grade 3–5 AEs.

## Graphical abstract

With 32 trials included in this research, we explored the clinical benefit and safety associated with mRNA vaccines for advanced solid tumors. The pooled ORR and DCR were 10.0% and 34.6%, respectively. The estimates for 1‐year and 2‐year PFS were 38.4% and 20.0%. The estimates for 1‐year and 2‐year OS were 75.3% and 45.5%. Grade 3–5 AEs were only 1.0%.

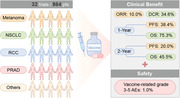

## 1 INTRODUCTION

Significant progress has been achieved in recent decades to cure cancer. Besides surgery, chemotherapy, and radiation as primary antitumor treatments, targeted therapies and immunotherapies have recently broadened and supplemented antitumor therapy.^1^ Recently, mRNA‐based cancer vaccines, exhibiting beneficial outcomes regarding modification and productivity, adjustable half‐time, and little gene integration risk, have become an innovative type of therapeutics. The knowledge derived from the context of COVID‐19 vaccine development leveraged to further development of mRNA‐based cancer immunotherapies.

mRNA cancer vaccines typically encode the entire or partial sequence of tumor‐specific antigens or tumor‐associated self‐antigens.^2^ After the injection of an mRNA vaccine, the encoded protein will be translated and transported to the immune system, which stimulates cellular and/or humoral immune responses and specifically eliminates malignant cells.^3^

Treating cancer patients using vaccines that stimulate an immune response is conceptually appealing. Importantly, unlike current vaccination strategies (such as DNA vaccines), the production of mRNA is faster, more flexible, and less expensive and it can be used for specific and individualized therapies.^4^ The utility of mRNA‐based cancer vaccination was first introduced by Conry et al. over 20 years ago.^5^ Several preclinical and clinical studies have been conducted since then. Basically, tumor mRNA vaccines are either generated (i) using ex vivo‐loaded dendritic cells (DCs). DCs can internalize RNAs by endocytosis and this process can be enhanced by electroporation. And then reinfusion of the transfected cells to patients via the intradermal, intranodal, or intravenous routes; (ii) by direct injection of mRNA with or without a carrier; carriers aid to promote stability, RNA uptake, and translatability of vaccines.^3^ Several carriers have been implemented, such as lipid nanoparticles (LNPs), lipoplexes (LPX), and protamine.^6^, ^7^, ^8^

To date, mRNA‐based cancer vaccination is being prevalently studied in clinical trials across a range of malignancies and has yielded mixed results. Thus, we conducted systematic research to quantitatively combine the results and seek to better assess the efficacy and safety by pooling published prospective studies, in which patients with progressed solid tumors received mRNA vaccines either as monotherapies or as combined choices.

In summary, we employed data analysis tools to synthesize the included clinical trial data. Clinical benefit (objective response rate [ORR], disease control rate [DCR], 1‐year/2‐year progression‐free survival [PFS], and overall survival [OS]) and safety (vaccine‐related grade 3−5 adverse events [AEs]) were investigated. Conclusively, mRNA vaccines appear to demonstrate modest clinical response rates, with acceptable survival rates and rare grade 3−5 AEs. Cancer types and delivery routes might determine the source of heterogeneities.

## 2 RESULTS

### 2.1 Study characteristics

**FIGURE 1:**
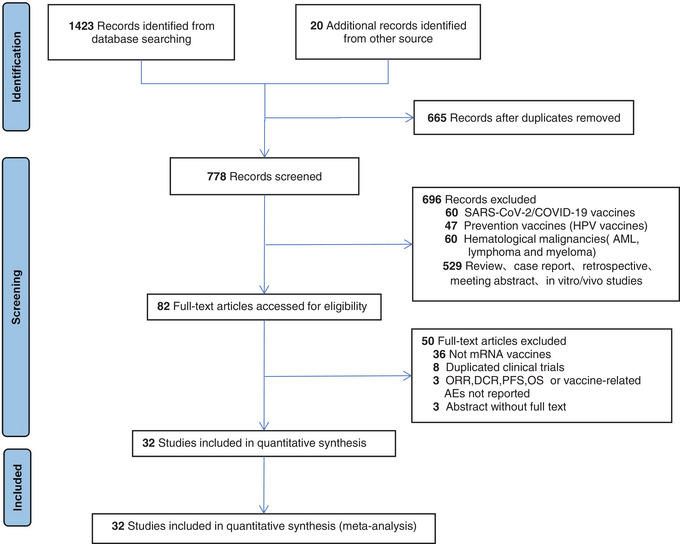
Preferred reporting items for systematic reviews and meta‐analyses flow diagram.

Thirty‐two prospective clinical trials comprising 984 patients with advanced solid tumors who had also undergone mRNA vaccines either as monotherapies or as combined choices were included in the meta‐analysis (Figure 1). The trials were published from 2002 to 2023 and the characteristics and findings of these trials were expressed in Table 1.^4^, ^9^, ^10^, ^11^, ^12^, ^13^, ^14^, ^15^, ^16^, ^17^, ^18^, ^19^, ^20^, ^21^, ^22^, ^23^, ^24^, ^25^, ^26^, ^27^, ^28^, ^29^, ^30^, ^31^, ^32^, ^33^, ^34^, ^35^, ^36^, ^37^, ^38^ These clinical trials consisted of 15 phases 1, 11 phases I/II, 5 phase II, and 1 phase III trial. There were 680 males (69.1%) and 304 females (30.9%). The median age was 58.0 years (interquartile range [IQR], 51.0−62.6 years) and the median follow‐up period was 28.5 months (IQR, 21.0−36.0 months). Among them, 30.4% of patients (*n* = 299) received mRNA vaccines as monotherapy and 69.6% (*n* = 685) received combined strategies. The most typical cancer types were melanoma (13 trials, 40.6%), non‐small cell lung cancer (NSCLC, 5 trials, 15.6%), renal cell carcinoma (RCC, 4 trials, 12.5%), and prostate adenocarcinoma (PRAD, 4 trials, 12.5%). The most prevalent delivery methods were ex vivo DCs loaded (22 trials, 68.7%), followed by direct injection of stabilized (8 trials, 25.0%) or naked (2 trials, 6.3%) RNA into lymph nodes, subcutaneous, muscle, or venous.

**TABLE 1:** Characteristics of included clinical trials.

												Clinical efficacy			PFS			OS			
															
Study	Trial number	No. of pts	Phase	Cancer types	Delivery (Route)	mRNA	Combination	No. of vaccines per pts, median (range)	Age, median (range), y	Sex (M/F)	Follow‐up, median (range), mo.	No./CR/PR/SD	ORR	DCR	1‐y	2‐y	Median, 95%CI, mon.	1‐y	2‐y	Median, 95%CI, mon.	Vaccine‐related grade 3−5 AEs
Heiser/2002	NR	13	I	Prostate cancer	DCs (i.d.)	PSA	None	NR	63 (49–76)	13/0	NR	NR	NR	NR	NR	NR	NR	NR	NR	NR	0
Su/2003	NCT00006431	10	I	Renal cell carcinoma	DCs (i.d. + i.v.)	Total tumor RNA	Adjuvant therapies	NR	57 (40–83)	9/1	11.6 (2–26.2)	NR	NR	NR	NR	NR	NR	NR	NR	Not reached	0
Caruso/2004	NR	7	I	Brain cancer	DCs (i.d. + i.v.)	Total tumor RNA	None	NR	15.5 (9–19.5)	1/6	NR	7/0/1/2	14%	43%	NR	NR	NR	NR	NR	NR	0
Caruso/2005	NR	7	I	Neuroblastoma	DCs (i.d. + i.v.)	Total tumor RNA	None	3 (3–5)	3.9 (3.4–5.8)	4/3	21 (14–36)	4/0/0/0	0%	0%	100%	0%	17.0 (15.8–18.2)	100%	16.7%	21.0 (19.9–22.1)	0
Mu/2005	NR	19	I/II	Prostate cancer	DCs (i.d./i.n.)	Total tumor RNA	None	4 (4–6)	69 (48−78)	19/0	NR	NR^a^	NR^a^	NR^a^	NR	NR	NR	NR	NR	NR	0
Weide/2008	NR	15	I/II	Melanoma	Naked (i.d.)	Total tumor RNA	GM‐CSF	7 (1–10)	48 (31–74)	8/7	NR	6/0/0/1	0%	17%	NR	NR	NR	NR	NR	NR	0
Weide/2009	NCT00204607	21	I/II	Melanoma	Protamine (i.d.)	Melan‐A, Tyrosinase, gp100, Mage‐A1, Mage‐A3, Survivin	GM‐CSF	11 (4–46)	60 (24–74)	10/11	NR	7/1/0/0	14%	14%	NR	NR	NR	75%	55%	25 (11.9–38.1)	0
Lesterhuis/2010	NCT00228189	5	I/II	CRC	DCs (i.d./i.v.)	CEA	None	NR	NR	NR	NR	NR	NR	NR	NR	NR	NR	NR	NR	NR	0
Wilgenhof/2011	NR	35	I	Melanoma	DCs (i.d.)	CD40L, CD70, TLR4, gp100, Tyrosinase, MAGE‐A3, MAGE‐C2	IFN‐a‐2b	NR	46 (28−75)	19/16	31 (20–40)	17/0/1/5	6%	35%	59.6%^b^	46.6%^b^	23 (1.4–44.7) ^b^	100%^b^	93.6%^b^	Not reached^b^	0
															14.9%^c^	9.8%^c^	3.11 (2.6–3.6)^c^	70%^c^	30%^c^	15.1 (9.4–20.9)^c^	
Vik‐Mo/2013	NCT00846456	7	I	Glioblastoma	DCs (i.d.)	hTERT, Survivin, CSCs	TMZ	NR	57 (46–63)	4/3	NR	7/0/0/0	0%	0%	85.7%	28.5%	23.1	100%	71.50%	25.3	0
Wilgenhof/2013	NCT01066390	15	IB	Melanoma	DCs (i.d. + i.v.)	CD40L, CD70, TLR4, gp100, Tyrosinase, MAGE‐A3, MAGE‐C2	None	NR	51 (41–78)	10/5	28 (22–34)	15/2/2/4	27%	53%	36.7%	36.7%	5 (0–10)	55.8%	36.7%	14 (5–23)	0
Amin/2015	NCT00678119	22	II	Renal cell carcinoma	DCs (i.d.)	CD40L, total tumor RNA	Sunitinib	6 (2–15)	56 (22–68)	16/5	NR	21/0/9/4	43%	62%	47.61%	19.0%	11.2 (6.0–19.4)	67.7%	53.0%	30.2 (9.4–57.1)	0
Bol/2015	NCT01530698	14	I/II	Melanoma	DCs (i.n.)	CD40L, CD70, caTLR4, gp100, Tyrosinase	None	3 (3–9)	54 (34–71)	6/8	NR	8/0/0/2	0%	25%	NR	NR	NR	NR	NR	NR	0
Kübler/2015	EudraCT number 2008‐003967‐37	44	I/IIa	Prostate cancer	Protamine (i.d.)	PSA, PSCA, PSMA, STEAP1	None	NR	67 (51–84)	44/0	NR	2/0/0/0	0%	0%	NR	NR	1.8 (1.4–3.2)^d^	84.2%	62.4%	29.3 (21.2–∞)	3
Kyte/2016	NCT01278940	22	I/II	Melanoma	DCs (i.d./i.n.)	Total tumor RNA	None	NR	57.5 (27–72)	9/13	NR	21/0/0/2	0%	10%	36.4%	21.2%	8.0 (4.2–11.8)	43%	23%	10	0
		9					IL‐2	16 (7–18)	58 (35–76)	6/3	NR	9/0/1/1	11%	22%	NR	NR	NR			13	0
Rittig/2016^j^	NR	14	I/II	Renal cell carcinoma	Naked (i.d.)	MUC1, CEA, Her2/neu, Telomerase, Survivin, MAGE‐A1	GM‐CSF	11 (4–32)	64.4 (36−79)	11/3	NR	30/0/1/15	3%	53%	18.7%	0%	2	70.1%	53%	24.5	0
		16							62.6 (44−73)	11/5					21.5%	7%	4				
Wilgenhof/2016	NCT01302496	39	II	Melanoma	DCs (i.d. + i.v.)	CD40L, CD70, TLR4, gp100, Tyrosinase, MAGE‐A3, MAGE‐C2	Ipilimumab	NR	46^e^ (24–70)	23/16	36 (22–43)	39/8/7/6	38%	54%	33%	22%	6.75 (2.25–11)	59%	38%	14.75 (10–19.75)	0
Kongsted/2017	NCT01446731	21	II	Prostate cancer	DCs (i.d.)	PSA, PAP, Survivin, and hTERT	Docetaxel	12 (7−18)	70 (60–84)	21/0	46.3	11/0/1/2	9%	27%	0%	0%	5.7 (1.7–9.7)	NR	NR	NR	1
Sahin/2017	NCT02035956	13	I	Melanoma	DCs (i.n.)	Personalized 20 neoantigens	None	18 (8–20)	61 (36–86)	8/5	(12–23)	5/1/1/1	40%	60%	84.6%	75.3%	NR	NR	NR	NR	0
Mitchell/2015	NCT00639639^f^	6	I	Melanoma	DCs (i.d.)	cmvpp65‐LAMP	STD‐TMZ + unpulsed DC	NR	50.5 (28–66)	2/4	NR	NR	NR	NR	16.2%	0%	10.8	49.8%	16.4%	18.5 (13.8–41.3)	0
		6	I	Melanoma	DCs (i.d.)	cmvpp65‐LAMP	STD‐TMZ + Td	NR	65 (30–75)	3/3	NR	NR	NR	NR	66.6%	50%	Not reached	100%	50%	41.4 (20.6–∞)	0
Batich/2017		11	I	Melanoma	DCs (i.d.)	cmvpp65‐LAMP	DI‐TMZ + GM‐CSF	NR	55 (47–67)	8/3	NR	NR	NR	NR	72.9%	54.7%	25.3 (11.0–∞)	100%	63.6%	41.1 (21.6–113.3)	0
Gururangan/2018	NCT01326104	10	I	Brain cancer	DCs (i.d.)	Total tumor mRNA and ex vivo expanded lymphocytes	None	3 (3–9)	NR	5/5	21	NR	NR	NR	NR	NR	5	NR	NR	13	1
Papachristofilou/2019	NCT01915524	26	Ib	NSCLC	Protamine (i.d.)	NY‐ESO‐1, MAGE‐C1, MAGE‐C2, Survivin, 5T4, and MUC‐1	Local radiation ± Pemetrexed/EGFR‐TKI	8.4 (2−25)	63.0 (40−83)	13/13	NR	26/0/1/12	4%	50%	15.1%	10%	2.87 (1.43–4.27)	61.4%	29.5%	13.95 (8.93–20.87)	3
Sebastian/2019	NCT00923312	46	I/II	NSCLC	Protamine (i.d.)	NY‐ESO‐1, MAGE‐C1, MAGE‐C2, Survivin, 5T4	None	NR	64.7^e^	29/15	NR	29/0/0/9	0%	31%	15.6%	NR	5 (1.8–6.3)	48.9%	30.9%	11.5 (8.5–18.8)	3
Cafri/2020	NCT03480152	4	I/II	Gastrointestinal cancer	RNA‐LNP (i.m.)	20 neoantigens	None	6 (4‐8)	45 (38–57)	2/2	NR	4/0/0/0	0%	0%	NR	NR	NR	NR	NR	NR	0
Boudewijns/2020	NCT02285413	11	II	Melanoma	DCs (i.d + i.v.)	Gp100, Tyrosinase	None^g^	NR	53 (25−69)	9/2	NR	NR	NR	NR	36.40%^i^	27.10%^i^	9.6^i^	63.50%	25.10%	32	0
		16					None^h^		61 (34−69)	8/8	NR	NR	NR	NR	6.2%	0%	3.0	75.0%	50.0%	19.0	0
		11					Cisplatin^g^		48 (25−67)	9/2	NR	NR	NR	NR	63.70%^i^	63.70%^i^	45.9^i^	100%	81.80%	Not reached	1
		16					Cisplatin^h^		54 (30−69)	10/6	NR	NR	NR	NR	6.2%	6.2%	4.7	56.2%	18.7%	12.2	
Figlin/2020	NCT01582672	307	III	Renal cell carcinoma	DCs (i.d.)	Tumor RNA, CD40L RNA	Sunitinib + SOC treatments	8	60	227/80	29 (0.4–47.7)	307/9/122/121	43%	82%	28.9%	15.1%	6 (5.8–6.7)	74.3%	53.4%	27.7 (23.0–35.9)	6
Jansen/2020	NCT01676779	21	II	Melanoma	DCs (i.d + i.v.)	CD40L, CD70, TLR4, gp100, Tyrosinase, MAGE‐A3, MAGE‐C2	None	NR	54 (24–81)	11/10	53 (3–67)	NR	NR	NR	71%	65.1%	NR	100%	90.2%	Not reached	0
Sahin/2020^k^	NCT02410733	42	I	Melanoma	RNA‐LPX (i.v.)	NY‐ESO‐1, Tyrosinase, TPTE, MAGE‐A3	None	NR	61 (21–86)	24/18	NR	25/1/3/7	16%	44%	NR	NR	NR	NR	NR	NR	NR
							Pembrolizumab/Nivolumab					17/0/6/2	35%	47%							
Palmer/2022	NCT03639714	14	I	GEA	RNA‐LNP (i.m.)	Personalized 20 neoantigens	Nivolumab + SC Ipilimumab	NR	59 (38–76)	10/4	NR	6/1/0/1	17%	33%	33%	17%	10.0	50%	17%	22.4	3
				NSCLC								1/0/0/0	0%	0%	0%	0%	1.8	0%	0%	17.4	
				CRC								7/0/0/3	0%	43%	14%	0%	5.4	43%	0%	8.7	
Ding/2022	NCT02956551	7	I	NSCLC	DCs (i.d.)	Personalized neoantigens	None	5 (3–14)	60 (47–73)	10/2	7.1 (0.9−17.2)	NR	NR	NR	NR	NR	2.2	NR	NR	7.6	0
		4					Nivolumab										11.2			11.2	
Gray^l^ /2022	NCT03164772	23	I/II	NSCLC	Protamine (i.d + i.v.)	NY‐ESO‐1, MAGE‐C1, MAGE‐C2, Survivin, 5T4, and MUC‐1	Durvalumab	12	64.9^e^	13/10	NR	19/0/5/7	26%	63%	NR	NR	2 (1.8–7.3)	NR	NR	23.5 (3.5–NA)	1
		34					Durvalumab + Tremelimumab	12	64.5^e^	10/24	NR	27/0/3/8	11%	41%			1.8 (1.2−2.8)			7.5 (3.4–10.5)	4

Abbreviations: AEs, adverse events; CI, confidence interval; CR, complete response; CRC, colorectal cancer; DCR, disease control rate; DCs, dendritic cells; DI‐TMZ, dose‐intensified temozolomide; GEA, gastroesophageal adenocarcinoma; GM‐CSF, granulocyte‐macrophage colony‐stimulating factor; i.d., intradermal; i.m., intramuscular; i.n., intranodal; i.v., intravenous; IFN‐a‐2b, interferon‐alpha‐2b; LNP, lipid nanoparticle; LPX, lipoplexes; M/F, male/female; NR, not applicable; NSCLC, non‐small cell lung cancer; ORR, objective response rate; OS, overall survival; PFS, progression‐free survival; PR, partial response; pts, patients; SD, stable disease; SOC, standard of care; STD‐TMZ, standard‐dose temozolomide; TD, tetanus/diphtheria; TKI, tyrosine kinase inhibitor.

^a^ PSA response were not included.

^b^ Patients without evidence diseases at baseline (*n* = 15).

^c^ Patients with evidence diseases at baseline (*n* = 20).

^d^ PSA‐progression‐free survival.

^e^ Data were presented as mean value.

^f^ Same NCT number with different populations.

^g^ Melanoma patients with stage III.

^h^ Melanoma patients with stage IV.

^i^ Recurrence free survival.

^j^ There were 14 patients in cohort A and 14 patients in cohort B.

^k^ Studies were not included in safety analysis because they did not specify vaccine‐related AEs.

^l^ The results of this clinical trial were published on the website (www.clinicaltrials.gov) and no relevant literature was retrieved to report the results.

### 2.2 Pooled ORR

**FIGURE 2:**
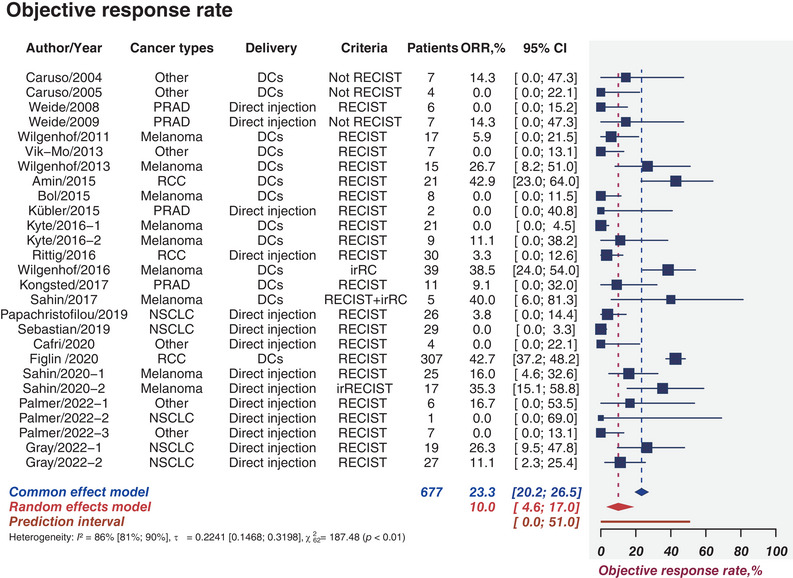
Clinical response. Sample sizes weighted random effects model was applied to depict forest plot of objective response rate related to tumor mRNA vaccines. PRAD, prostate adenocarcinoma; NSCLC, non‐small cell lung cancer; DCs, dendritic cells; RECIST, Response Evaluation Criteria in Solid Tumors; irRC, immune‐related response criteria; immune‐related response evaluation criteria in solid tumors; ORR, objective response rate; DCR, disease control rate; CI, confidence interval.

ORR is defined as the proportion of patients whose tumors have shrunk to predefined volumes and who are able to maintain minimum time requirements. Twenty‐two studies comprising 677 patients provided rates of ORR, which ranged from 0 to 42.9%. The pooled ORR was calculated as 10.0% (95%CI, 4.6−17.0%; *I* ^2^ = 86%; 95%CI, 81−90%; Figure 2). Figures S1A and S2A depict the Egger test and funnel plot of ORR with the *p* value of 0.03, signifying the presence of publication bias. Subgroup analyses were performed for various cancer types, delivery methods, and treatment modalities (Figures S3 and S4). When stratified by cancer types, nine, four, and three studies were accounted for in melanoma, PRAD, and RCC, respectively. The estimated ORR were 26.4% (95%CI, 0.0−92.6%) for RCC, 14.6% (95%CI, 3.5−31.6%) for melanoma, and 5.4% (95%CI, 0.0−25.3%) for PRAD (Figure S3). DCs delivery had a modestly higher ORR than direct injections (13.5 vs. 6.9%, *p* = 0.27; Figure S3) and integrated therapies had a modestly higher ORR than monotherapies (13.6 vs. 4.6%, *p* = 0.12; Figure S4).

In total, the pooled ORR was calculated as 10.0%. Regarding the subgroup analysis, the estimated ORR was greater than other groups in the group of RCC, DCs delivery, and combined therapies.

### 2.3 Pooled DCR

**FIGURE 3:**
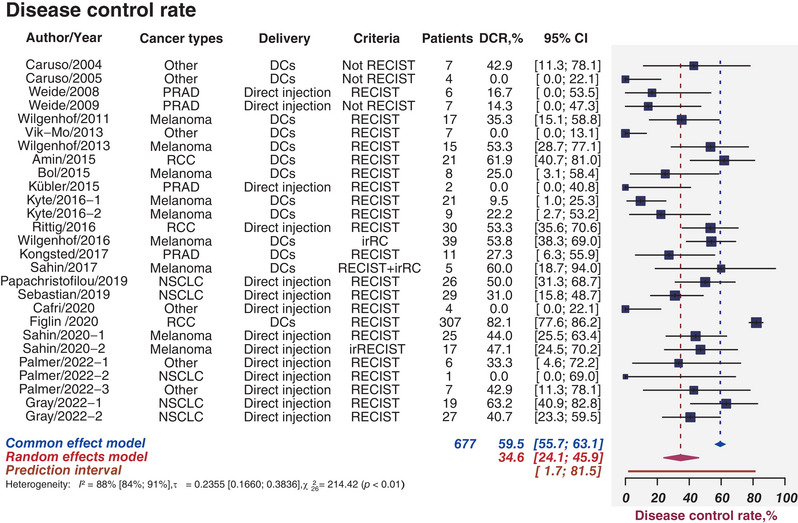
Clinical response. Sample sizes weighted random effects model was applied to depict forest plot of disease control rate related to tumor mRNA vaccines. PRAD, prostate adenocarcinoma; NSCLC, non‐small cell lung cancer; DCs, dendritic cells; RECIST, Response Evaluation Criteria in Solid Tumors; irRC, immune‐related response criteria; immune‐related response evaluation criteria in solid tumors; ORR, objective response rate; DCR, disease control rate; CI, confidence interval.

DCR is defined as the percentage of cases with remission and stable disease after treatment. Twenty‐two studies comprising 677 patients provided rates of DCR, which ranged from 0 to 82.1%. Figure 3 illustrates the forest plot for all 22 studies. The pooled DCR was estimated as 34.6% (95%CI, 24.1−45.9%; *I* ^2^ = 88%, 95%CI, 84−91%). Figures S2B and S7 depict the funnel plot and Egger test in which the *p* value was less than 0.01, denoting the presence of publication bias. When stratified by cancer types, the estimated DCR was 68.1% (95%CI, 27.3−96.8%) for RCC, 38.0% (95%CI, 24.7−52.3%) for melanoma, and 17.7% (95%CI, 2.8−41.4%) for PRAD (subgroup *p* < 0.01; Figure S5). The estimated DCR for DCs delivery and direct injections were 34.4 and 37.9%, respectively (subgroup *p* = 0.74; Figure S5). Combined therapies had modestly higher DCR than monotherapy (40.8 vs. 24.1%, *p* = 0.13; Figure S6).

In summary, the pooled DCR was determined as 34.6%. The determined DCR was higher than other groups in the group of RCC, direct injections, and combined therapies when we perform subgroup analysis.

### 2.4 1‐Year PFS

**FIGURE 4:**
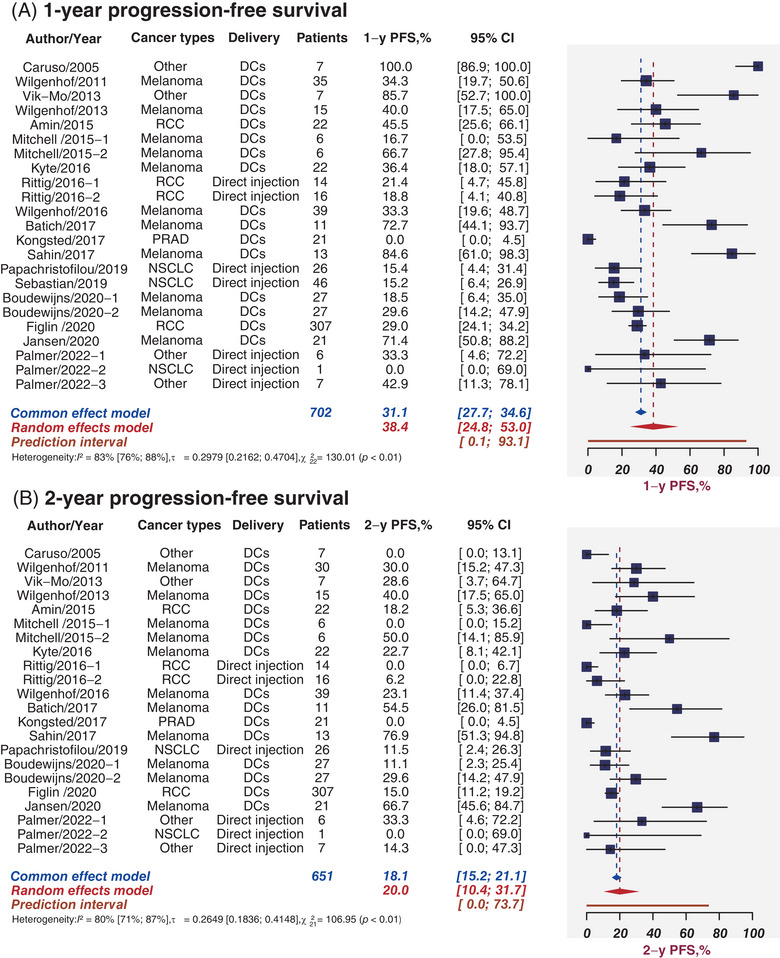
Progression‐free survival benefit. Sample sizes weighted random effects model was applied to depict forest plot of 1‐year (A) and 2‐year (B) progression‐free survival related to tumor mRNA vaccines. PRAD, prostate adenocarcinoma; NSCLC, non‐small cell lung cancer; DCs, dendritic cells; PFS, progression‐free survival; CI, confidence interval.

PFS refers to the time from randomization to the appearance of objective tumor progression or all‐cause death, which may indirectly reflect the clinical benefit of patients. Twenty‐one studies including 702 patients indicated the rates of PFS at 1 year, ranging from 0 to 100%. Figure 4A illustrates the forest plot for 1‐year PFS among all 21 studies. The estimated 1‐year PFS was 38.4% (95%CI, 24.8−53.0%; *I* ^2^ = 83%, 95%CI, 76−88%). Figures S7 and S1C depicted the corresponding funnel plot and Egger test in which the *p* value < 0.01, denoting the presence of publication bias. When stratified by cancer types, the pooled 1‐year PFS was 44.7% (95%CI, 28.9−61.0%) and 29.1% (95%CI, 20.8−38.2%) for melanoma and RCC, respectively (Figure S8). DC vaccines tended to have higher 1‐year PFS (46.2%, 95%CI, 27.6−65.4%) than direct injections (18.5%, 95%CI, 11.7−26.3%, subgroup *p* < 0.01; Figure S8). The pooled 1‐year PFS was 54.2% and 31.5% for monotherapy and combined therapies, respectively (subgroup *p* = 0.18; Figure S8).

Overall, the pooled 1‐year PFS was calculated as 38.4%. For the subgroup analysis, the estimated 1‐year PFS was higher than other groups in the group of melanoma (44.7%), DCs delivery (46.2%), and combined therapies (54.2%).

### 2.5 2‐Year PFS

Twenty studies comprising 651 patients provided the rates of PFS at 2 years, ranging from 0 to 76.9%. The estimated 2‐year PFS was 20.0% (95%CI, 10.4−31.7%; *I* ^2^ = 80%, 95%CI, 71−87%) depicted in Figure 4B. Figures S7 and S1D illustrated the corresponding funnel plots and Egger test in which the *p* value < 0.01, identifying the presence of publication bias. The pooled 2‐year PFS declined to 34.1% (95%CI, 17.7−52.6%) and 8.6% (95%CI, 0.0−31.6%) for melanoma and RCC, respectively (Figure S10). DC vaccines also tended to have higher 2‐year PFS (24.5%, 95%CI, 11.8−39.9%) than direct injections (7.8%, 95%CI, 0.4−23.0%, subgroup *p* = 0.04; Figure S10). The pooled 2‐year PFS was 31.9% and 15.7% for monotherapy and combined therapies, respectively (subgroup *p* = 0.26; Figure S11).

In total, the pooled 2‐year PFS was estimated as 20.0%. The estimated 2‐year PFS was greater than other groups in the group of melanoma (34.1%), DCs delivery (24.5%), and monotherapy therapies (31.9%) when we perform subgroup analysis.

### 2.6 1‐ and 2‐Year OS

**FIGURE 5:**
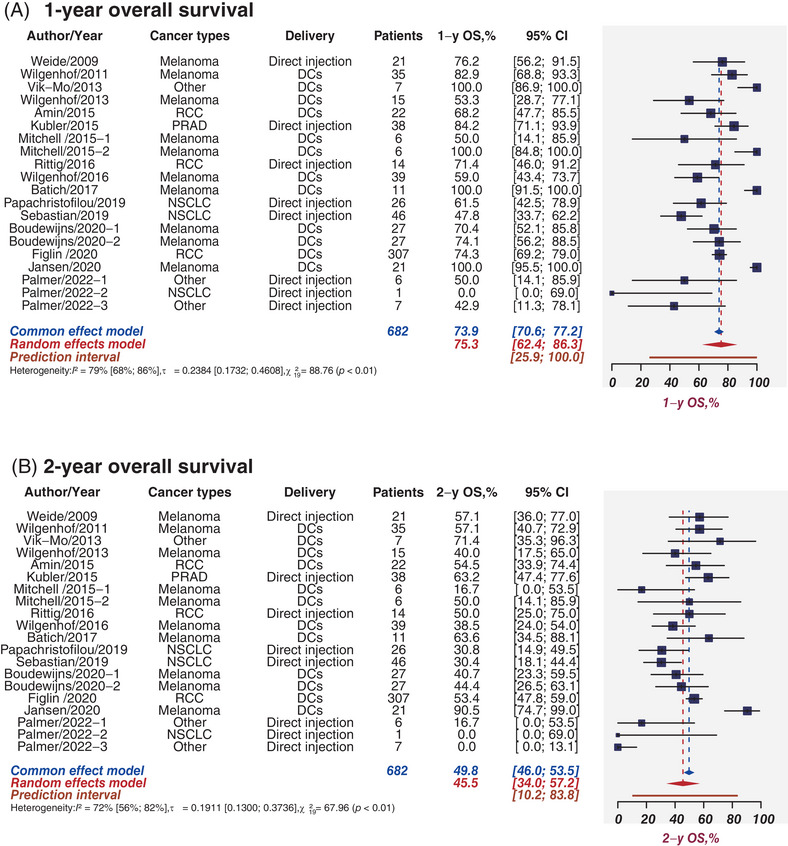
Overall survival benefit. Sample sizes weighted random effects model was applied to depict forest plot of 1‐year (A) and 2‐year (B) overall survival related to tumor mRNA vaccines. PRAD, prostate adenocarcinoma; NSCLC, non‐small cell lung cancer; DCs, dendritic cells; OS, overall survival; CI, confidence interval.

OS refers to the time from randomization to death from any cause. Seventeen studies comprising 682 patients provided the rates of OS at 1 and 2 years, ranging from 41.9% to 100% and 14.3% to 90.5%, respectively. Figure 5 expressed the pooled results of 17 studies and the estimated 1‐year OS and 2‐year OS were 75.3% (95%CI, 62.4%−86.3%; I^2^ = 79%, 95%CI, 68%−86%) and 45.5% (95%CI, 34.0−57.2%; *I* ^2^ = 72%, 95%CI, 56−82%), respectively. The Egger test and funnel plots revealed the absence of publication bias for 1‐year OS (*p* = 0.20) or 2‐year OS (*p* = 0.53; Figures S1E, F and S12). Regarding 1‐year OS, the pooled 1‐year OS estimates were 82.2% for melanoma and 73.8% for RCC (Figure S13); 83.1% for DCs and 63.2% for direct injections (subgroup *p* = 0.05; Figure S13); 76.2% for monotherapies and 75.1% for combination therapies (subgroup *p* = 0.94; Figure S14). Regarding 2‐year OS, the pooled 2‐year OS estimates were 51.5% for melanoma and 53.4% for RCC (Figure S15); 52.7% for DCs and 31.9% for direct injections (subgroup *p* = 0.09; Figure S15); 53.9% for monotherapy and 42.9% for combination therapies (subgroup *p* = 0.41; Figure S16).

Overall, the pooled 1‐year OS was determined as 75.3%. The estimated 1‐year OS was greater than other groups in the group of melanoma (82.2%), DCs delivery (83.1%), and monotherapy therapies (76.2%) when we perform subgroup analysis. For 2‐year OS, the pooled outcomes were estimated as 45.5%. The estimated 2‐year OS was greater than other groups in the group RCC (53.4%), DCs delivery (52.7%), and monotherapy therapies (53.9%).

### 2.7 Vaccine‐related grade 3−5 AEs

**FIGURE 6:**
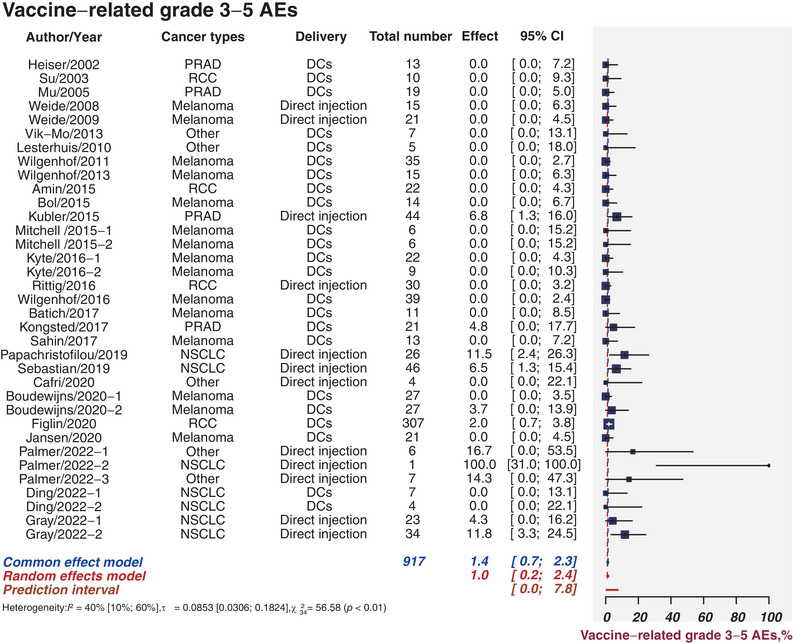
Safety analysis. Sample sizes weighted random effects model was applied to depict forest plot of grade 3−5 adverse events related to tumor mRNA vaccines. PRAD, prostate adenocarcinoma; NSCLC, non‐small cell lung cancer; DCs, dendritic cells; AEs, adverse events; CI, confidence interval.

The safety of vaccines is usually assessed by grade 3–5 vaccine‐related AEs. Twenty‐eight studies involving 917 patients signified the prevalence of vaccine‐related grade 3−5 AEs, which ranged from 0.0 to 100.0%. Twenty‐two studies (78.6%) reported zero grade 3−5 AEs. Figure 6 illustrated the estimated incidence was 1.0% (95%CI, 0.2−2.4%; *I* ^2^ = 40%, 95%CI, 10−60%). There were nine studies^22^ ^;^ ^28^, ^29^, ^30^, ^31^, ^32^ ^;^ ^34^ ^;^ ^38^ that reported grade 3−5 AEs linked to vaccines (Table S1). The funnel plot revealed the tiny publication bias (*p* = 0.04; Figure S17).

In general, the adverse effects of the tumor vaccine are generally acceptable (the estimated incidence was 1.0%). And the findings of sensitivity analyses have been illustrated in Figures S18–S23.

## 3 DISCUSSION AND CONCLUSION

This research comprising thirty‐two prospective clinical trials revealed that mRNA vaccines tend to exhibit modest clinical response rates; the pooled ORR and DCR estimates were 10.0 and 34.6%, respectively. Survival rates (1‐year PFS 38.4%, 2‐year PFS 20.0%; 1‐year OS 75.3%, 2‐year OS 45.5%) are acceptable, with significantly rare grade 3−5 AEs (1.0%). To the best of our knowledge, this is the only meta‐analysis assessing the mRNA vaccines as a therapeutic prospectively administered in the aspect of advanced solid tumors. Our analysis highlighted pooled statistics of clinical response rates, survival rates, and incidence of high‐grade AEs linked to mRNA vaccines. Our outcomes are significant in assisting clinical trial design and providing implications for clinical practice. The heterogeneity test results reveal high heterogeneity overall, which may be because multiple tumors (even different immune subtypes of the same tumor) typically have different immune characteristics, usually expressed as immunologically “Hot” and “Cold” tumors. Therefore, the level of response to immunotherapy or immune‐gene therapy varies significantly. However, when patients are recruited in clinical trials, we can only discern the pathological type or progression stage of the tumor, and it is difficult to make immunological differentiation. This also induces discouraging heterogeneity in the treatment of tumors estimated in general. Therefore, assessing the candidate tumor antigen of mRNA vaccine and identifying the proper patient for vaccination are the major links in the development of tumor mRNA vaccine in the future.

It should be known that the pooled estimate for ORR was only 10%. These data do not imply that mRNA vaccines could not be applied in clinical practice. The highest ORR was found in Amin et al. (42.9%)^20^ and Figlin et al. (42.7%).^34^ The mutual characteristics of the two studies were that they all focused on RCC, used ex vivo DC loading, and applied combination modalities. In Amin's research, the median PFS was 11.2 months and the median OS was 30.2 months. This implies that the expected survival rate of this group of patients has almost doubled.^28^ The median OS was estimated to be more than 5 years, which implies that in intermediate‐risk mRCC patients, a combination of targeted therapy and immunotherapy may have a great impact on survival outcomes. The mechanism of AGS‐003 is to influence CD8+, CD28+, and CD45RA−CTLs against patient‐specific tumor antigens. To influence effective CD8+cytotoxic T‐cell response, activated helper CD4 + T cells up‐regulate CD40L and interact with DCs. Due to systemic and local facilitators of the tumor cells, mRCC patients are immunosuppressed, which demonstrates DC and CD4+helper T‐cell dysfunction. To address that, RNA encoding CD40L was coelectroporated into ex vivo prepared DCs to simulate the presence of CD4+ T cells by intracellular ligation of endogenous CD40 in DCs. This has been linked to its good clinical outcomes in participants with solid tumors. This is probably why the DCs method performed better in most of the results.

Cancer mRNA vaccines for therapy necessitate substantial doses and strong vaccine potency to facilitate tumor responses. Monotherapy is usually employed for some early‐stage cancers or only as a subordinate treatment. However, for advanced malignant tumors, it seems difficult to have a promising effect if monotherapy was employed. Integrating mRNA cancer vaccines with other immunotherapeutic approaches, such as adoptive cell therapy, immune checkpoint inhibitors, and oncolytic viruses, may be able to demonstrate considerable clinical response in the treatment of malignant tumors in the future. While elevating clinical response rates and PFS, lower toxicity and side effects are also significant considerations. Our subgroup analysis further verified the preceding findings. More importantly, studies have indicated that the ORR did not always correlate with 1‐year OS.^39^ The correlation coefficient (*r*) of ORR with 1‐year OS was only 0.08 (poorly calibrated) in checkpoint‐inhibitor therapies. Despite mRNA vaccines resulting in relatively low ORR rates in advanced solid tumors, the 1‐year OS linked to mRNA vaccines could reach 75.3% (95%CI, 62.4−86.3%), which was similar to other immunotherapies such as programmed death 1 (PD‐1) and programmed death ligand 1 (PD‐L1) inhibitors, as prior meta‐analysis has indicated that the 1‐year OS linked to atezolizumab (PD‐L1 inhibitor)^40^ and nivolumab (PD‐1 inhibitor)^41^ were 55% (95% CI, 49−61%) and 52% (95% CI, 43−62%) in malignancies, respectively. Furthermore, the selection of target antigens can be a major factor impacting the efficacy of mRNA vaccines. Typically, nonmutated self‐antigens (so‐called “public” antigens) or mutated neoantigens are chosen. Regarding nonmutated self‐antigens, as tumor antigens are acquired from self‐antigens, it is probable that high‐avidity T‐cell receptors will have been eliminated from the repertoire, compromising efficacy.^42^ Malignant cells always harbor mutations and are often genetically unstable, leading to several alterations in the repertoire of epitopes (neoantigens) they present. Mutated neoantigens are promising vaccine targets as they are ample in many tumor types and highly tumor specific.^43^ The first‐in‐human application of this concept in melanoma was demonstrated by Sahin et al.^4^ with promising outcomes (ORR 40%, DCR 60%, 1‐y PFS 84.6%). As demonstrated in this neoantigen clinical trial, one complete response has been revealed when combining neoantigen mRNA vaccines with PD‐1 blockade therapies, which inspires the integration of mRNA vaccines and immune checkpoint inhibitors. To date, several clinical trials have been performed to investigate these combination modalities^44^ and their results are still unknown.

Another major factor that may influence vaccine effectiveness is the route of delivery.^42^ The route of administration ascertains the distribution of the vaccine and eventually affects its efficacy. Because of the large proportion of subcutaneously antigen‐presenting cells, intradermal and subcutaneously injected mRNA vaccines are readily delivered to yield corresponding immune responses. But this technique usually influences a considerable local injection‐site response. intranodal administration of mRNA reaches the lymph antigen‐presenting cell directly. But it is cumbersome and the volume of the injection is restricted. For the intramuscular injection, the blood supply to the muscle tissue is sufficient and, in general, fewer injection‐site reactions are induced. Intravenous injection enables the mRNA vaccine to be delivered throughout the body, and this administration has been found to facilitate a robust CD8+ T‐cell response. Intravenous administration is therefore the most common direct route of administration in mRNA cancer vaccine trials.^45^ Furthermore, the optimal timing of vaccine application (early vs. advanced disease), manipulation of the tumor microenvironment, and potential combination strategies that may promote additive or even synergistic antitumor effects must be considered.^46^, ^47^, ^48^, ^49^

Short‐term side effects of the mRNA vaccine in the treatment of malignant tumors encompass fever, muscle pain, headache, joint pain, nausea, vomiting, diarrhea, and rash. Long‐term side effects may comprise immune responses including anaphylaxis, chronic inflammation, and autoimmune reactions. The mechanism of these side effects is primarily due to the immune response influenced by the mRNA vaccine, which may lead to the extreme immune response of the body, thus leading to the occurrence of the preceding side effects. Among these, cytokine release syndrome is one of the most concerned.^22^ The incidence of grade 3−5 AEs linked to mRNA vaccines seems low (less than 1%), which implies that the application of mRNA vaccines in clinical settings was safe and they might be appropriate for adjuvant therapies without worrying about the added toxicities. However, low incidence does not indicate that it would not occur. One possibly vaccine‐related death was reported by Kübler et al.^22^ 1 month following the 4th vaccination. Furthermore, cytokine release syndrome has also been found in a patient with colorectal cancer 5 days after administration of COVID‐19 mRNA vaccines with long‐standing anti‐PD‐1 monotherapy.^50^ This case report also reminds us of the significance of surveillance and control of AEs when integrating mRNA vaccines with other immunotherapies.

Our research has limitations. First, only published prospective clinical trials were included in our analyses, with the presence of publication bias. Second, we could not access individual patient data. Therefore, we were unable to control for significant patient‐specific covariates that might influence the outcomes of our research. Third, the pooled analysis presented evidence in several cancer types, different delivery vehicles and routes, and treatment modalities, while the sources of high heterogeneity have not been fully identified. Future large prospective trials should fixate on these factors and optimize the efficacy and mitigate the adverse effects of tumor mRNA vaccines in clinical settings.

Conclusively, active immunotherapy is forthcoming as a crucial addition to traditional cancer treatments. In this systematic review and meta‐analysis, mRNA vaccines seemed to be safe and promising in specific types of advanced solid tumors. Many factors could influence the effectiveness of mRNA vaccines. Widespread efforts must be made to assess optimal combinations of antigens, adjuvants, and delivery vehicles. Predictive biomarkers that can determine subpopulations of patients are most likely to be the focus of future cancer vaccine analysis. And more personalized treatment will also be a critical direction for future therapeutic cancer vaccines.

## 4 METHODS AND MATERIALS

### 4.1 Search strategy and study selection

A systematic search of electronic databases including PubMed and Embase between January 1, 2000 and January 4, 2023, with an English language barrier was executed according to Evidence Acquisition the Population, Intervention, Control, Outcomes, and Study Design. The Preferred Reporting Items for Systematic Reviews and Meta‐analyses reporting guideline was employed.^51^ The following keywords were employed in PubMed searching: “cancer,” “carcinoma,” “tumor”, “mRNA,” “messenger RNA,” “vaccine,” “vaccines,” and “vaccination.” Reviews were excluded but they were also tested for potential qualified research. The whole search was executed by three independent authors (T. Y. Z., H. X., and X. N. Z).

Prospective clinical trials were included if they met the following criteria: (1) Patients with advanced solid tumors; (2) Investigating mRNA vaccines either as monotherapies or as combined choices; (3) single or multiarm studies; (4) reporting at least one of the following findings (ORR; DCR; 1‐year and/or 2‐year PFS; 1‐year and/or 2‐year OS; the vaccine‐related grade 3−5 AEs. Exclusion criteria included: (1) not mRNA vaccines; (2) vaccines for prevention use; (3) in hematological malignancies settings; (4) review, case report, retrospective, in vitro/vivo studies; (5) not provided any of the outcomes; (6) were expressed on meeting abstracts without full text published. The selection flow diagram is depicted in Figure 1. If two or more studies presented results using the same populations, only the most thorough (which provided our outcomes), relevant and latest study was included in the analysis. Disparities were addressed by discussion.

### 4.2 Outcome measures and data extraction

The main outcomes were ORR and DCR. The secondary outcomes were 1‐year and/or 2‐year PFS, 1‐year and/or 2‐year OS, and the vaccine‐related grade 3−5 AEs.

Data extraction was undertaken by two authors (T. Y. Z. and H. X.) and evaluated by a third author (S. Y. Z). Trial details, treatment characteristics, and outcomes were computed and expressed in Table 1. Data were extracted from the arm or cohort level. Rates of grade 3–5 AEs were largely based on the Common Terminology Criteria for Adverse Events. If studies did not determine whether the AEs were vaccine related, they were not included in the safety analysis. The specific grade 3−5 AEs linked to mRNA vaccines were expressed in Table S1. Particularly, if one analysis did not present details on the basic information (such as median age and median vaccine doses), individual patient data highlighted in the main text or supplementary materials were applied to complete the calculation. Furthermore, if one study did not highlight the 1‐year or 2‐year PFS or OS in their text, we applied Plot Digitizer, version 2.6.8 (Source‐Forge) to extract the 1‐year and 2‐year survival rates from the Kaplan–Meier curves (where provided). The survival rates extraction process was executed by two authors (T. Y. Z. and H. X.) and pooled by another author (X. N. Z). Disparities were resolved by discussion.

As for each forest plot of certain survival rates, the cases were calculated by multiplying the proportion of patients included in the arms by the survival rates at that time point. The cases were rounded to the nearest integral number. For instance, if one study comprised 100 patients with 1‐year survival rates of 75.9%, then the cases were calculated as 76.

### 4.3 Statistical analysis

All analyses were executed using R Studio (version 1.4.1717) with R (version 4.1.0). the R packages (meta and metafor) were employed for all statistical analyses. Random effects models weighted by the number of patients were applied for all meta‐analyses as they supersede fixed effects models concerning treatment decisions. All models were fit using angular transformation and when an event was zero or one, a 0.5 continuity correction was applied.^52^ Besides, the limited maximum likelihood method with the Hartung–Knapp adjustment^53^ was applied. Heterogeneity was investigated using the Cochran *Q* statistic and *I* ^2^ statistics. A *p* value less than 0.05 was deemed statistically significant. Publication bias was visualized by funnel plots. Egger linear regression test was also employed to discover publication bias. Subgroup analysis was executed based on cancer types, delivery vehicles, and treatment modalities. R code for DCR subgroup analysis was available in Supplementary Method S1. We also conducted sensitivity analyses. By removing the smaller sample size trials (<10 patients), a minor difference was detected between the outcomes and the pooled effect (Figures S18–S20). Similarly, subgroup analysis of the year of publication also disclosed that overall outcomes did not vary considerably by removing clinical trials undertaken a decade prior (Figures S21–S23). Microsoft PowerPoint was used to draw the graphical abstract.

## AUTHOR CONTRIBUTION

Jian‐zhong Ai had full access to all of the data in the study and take responsibility for the integrity of the data and the accuracy of the data analysis. Tian‐yi Zhang, Hang Xu, and Xiao‐nan Zheng contributed equally to this work. *Concept and design*: J. Ai, H. Xu, and T. Zhang. *Acquisition, analysis, or interpretation of data*: All authors. *Drafting of the manuscript*: J. Ai, T. Zhang, H. Xu, and X. Zheng. *Critical revision of the manuscript for important intellectual content*: J. Ai, H. Xu, T. Zhang, S. Zhang, X. Xiong, and J. Li. *Statistical analysis*: H. Xu, X. Zheng, T. Zhang, X. Xiong, S. Zhang, and X. Yi. *Administrative, technical, or material support*: *Q*. Wei and J. Ai. *Supervision*: J. Ai. All authors have read and approved the final manuscript.

## CONFLICT OF INTEREST STATEMENT

The authors declare no conflict of interest.

## ETHICS STATEMENT

No ethics approval required.

## Supporting information

Supporting Information

## Data Availability

Not applicable.
