# Supplementary material for: Clinical benefit and safety associated with mRNA vaccines for advanced solid tumors: A meta‐analysis
Source: MedComm (2020). 2023 Jul 18;4(4):e286. doi: 10.1002/mco2.286 (PMC10353527; doi:10.1002/mco2.286)
Supplement: Supplementary file 1 — Supporting Information [file MCO2-4-e286-s001.pdf]

Supplementary information

Clinical benefit and safety associated with mRNA vaccines for advanced solid tumors: a meta-analysis

Authors:

Tian-yi Zhang 1, 2, #; Hang Xu 1, 2, #; Xiao-nan Zheng1, 2, #; Xing-yu Xiong1, 2 ; Shi-yu Zhang1, 2 ;Xian-yanling Yi1, 2 ; Jin Li1, 2; Qiang Wei 1, 2; Jianzhong Ai 1, 2, *

Affiliations:

1. Institute of Urology, West China Hospital, Sichuan University, Chengdu, China, 610041
2. Department of Urology, West China Hospital, Sichuan University, Chengdu, China, 610041

* **Corresponding authors**:

Jian-zhong Ai, PhD, Institute of Urology, Department of urology, West China Hospital, Sichuan University, Chengdu, China, 610041 (jianzhong.ai@scu.edu.cn)

# These authors contributed this study equally.

Table S1. Specific Grade 3-5 AEs related to tumor mRNA Vaccines

Figure S1. Egger test and Funnel plotfor publication bias

Figure S2. Funnel plot for publication bias regarding objective response rate (ORR) and disease control rate (DCR)

Figure S3. Forest plot of objective response rate (ORR), stratified by cancer types and vehicles

Figure S4. Forest plot of objective response rate (ORR), stratified by treatment modalities

Figure S5. Forest plot of disease control rate (DCR), stratified by cancer types and vehicles

Figure S6. Forest plot of disease control rate (DCR), stratified by treatment modalities

Figure S7. Funnel plot for publication bias regarding 1-year and 2-year progression-free survival (PFS)

Figure S8. Forest plot of 1-year progression-free survival (1-y PFS), stratified by cancer types and vehicles

Figure S9. Forest plot of 1-year progression-free survival (1-y PFS), stratified by treatment modalities

Figure S10. Forest plot of 2-year progression-free survival (2-y PFS), stratified by cancer types and vehicles

Figure S11. Forest plot of 2-year progression-free survival (2-y PFS), stratified by treatment modalities

Figure S12. Funnel plot for publication bias regarding 1-year and 2-year overall survival (OS)

Figure S13. Forest plot of 1-year overall survival (1-y OS), stratified by cancer types and vehicles

Figure S14. Forest plot of 1-year overall survival (1-y OS), stratified by treatment modalities

Figure S15. Forest plot of 2-year overall survival (2-y OS), stratified by cancer types and vehicles

Figure S16. Forest plot of 2-year overall survival (2-y OS), stratified by treatment modalities

Figure S17. Funnel plot for publication bias regarding vaccine-related grade 3-5 AEs

Figure S18. Forest plot of objective response rate (ORR) and disease control rate (DCR), clinical trials with a sample size of less than 10 patients were excluded

Figure S19. Forest plot of 1-year and 2-year progression-free survival (PFS),

clinical trials with a sample size of less than 10 patients were excluded

Figure S20. Forest plot of 1-year and 2-year overall survival (2-y OS),clinical trials with a sample size of less than 10 patients were excluded

Figure S21. Forest plot of objective response rate (ORR) and disease control rate (DCR), stratified by published year.

Figure S22. Forest plot of 1-year and 2-year progression-free survival (PFS), stratified by published year

Figure S23. Forest plot of 1-year and 2-yearoverall survival (OS), stratified by published year

Method S1. R Code for subgroup analysis

Table S1. Specific Grade 3-5 AEs related to tumor mRNA Vaccines

Study	AEs	CTCAE grade	Number	Days onset	Action taken
Kübler 2015	Bronchopneumonia	5	1	1 month after having received the 4th vaccination	NR
	Urinary retention	3	1	The second vaccination	Symptomatic and antibiotic treatment
	Urinary retention with hydronephrosis	3	1	The second vaccination	Symptomatic and antibiotic treatment
Kongsted 2017	Incidental finding of pulmonary embolisms	3-4	1	1 week after the leukapheresis procedure	NR
Gururangan 2018	Elevation of serum alkaline phosphatase	3	1	3 months after the 3rd dose of ttRNA DC	NR
Papachristofilou 2019	Fatigue	3	2	NR	NR
	Pyrexia	3	1	NR	NR
Sebastian 2019	Fatigue	3	1	NR	NR
	Injection site pustule	3	1	NR	NR

	Asthma	3	1	NR	NR
Boudewijns 2020	Decompensated heart failure	3	1	NR	NR
Figlin 2020	NR	3-5	6	NR	NR
Palmer/2022	Hyperthyroidism<br>Transaminases increased<br>Duodenitis	3-4	3	100 days after having received the vaccination	NR
Gray /2022	NR	3-4	5	90 days after the last dose of study treatment	NR

Abbreviations: AEs, adverse events; CTCAE, Common Terminology Criteria for Adverse Events; DC, dendritic cell; NR, not report

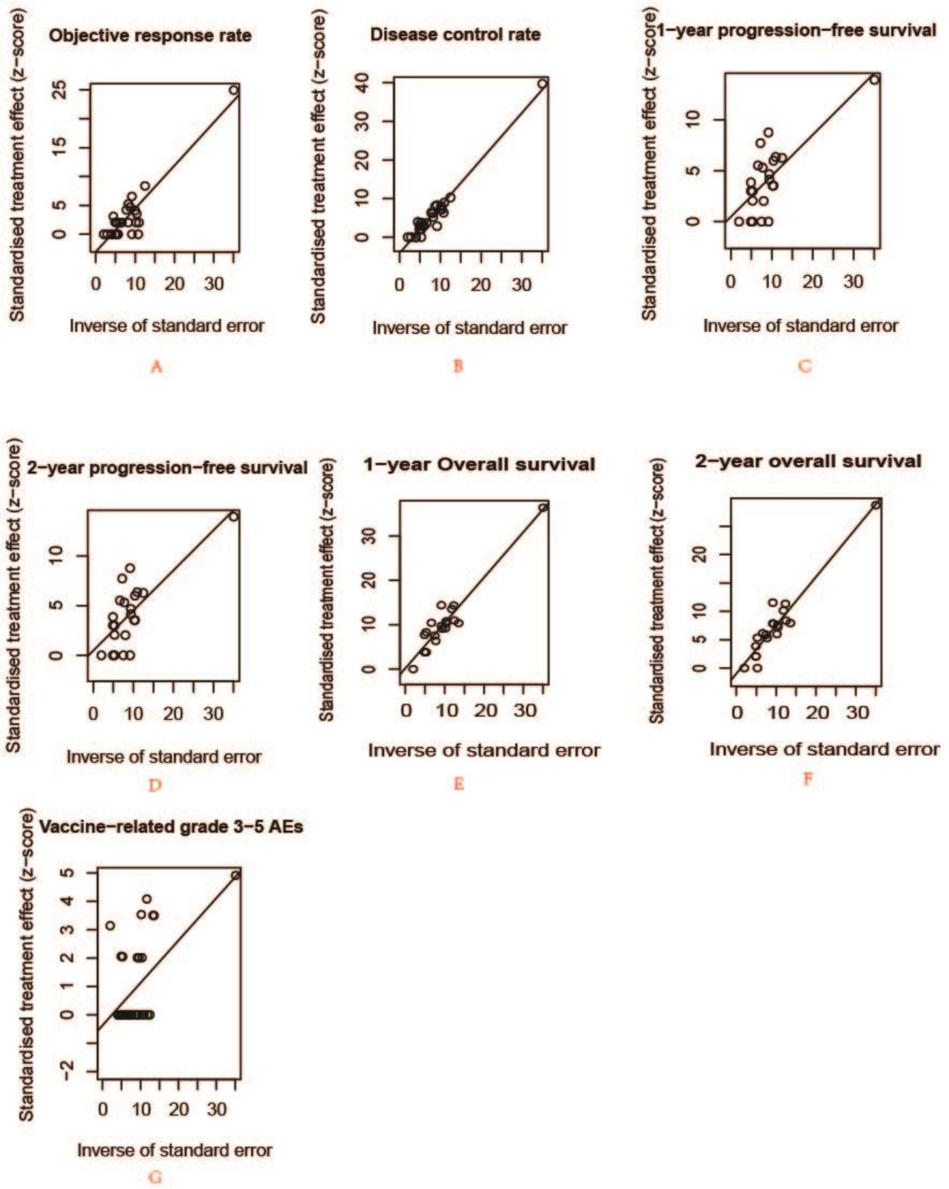

Figure S1. Egger test for publication bias

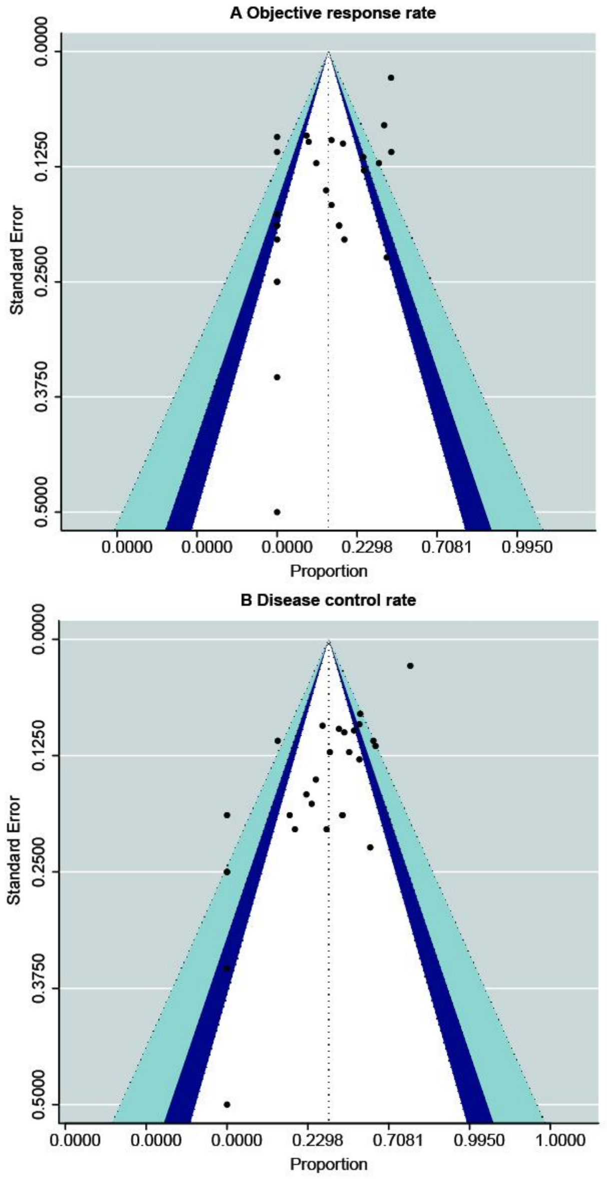

Figure S2. Funnel plot for publication bias regarding objective response rate (ORR) and disease control rate (DCR)

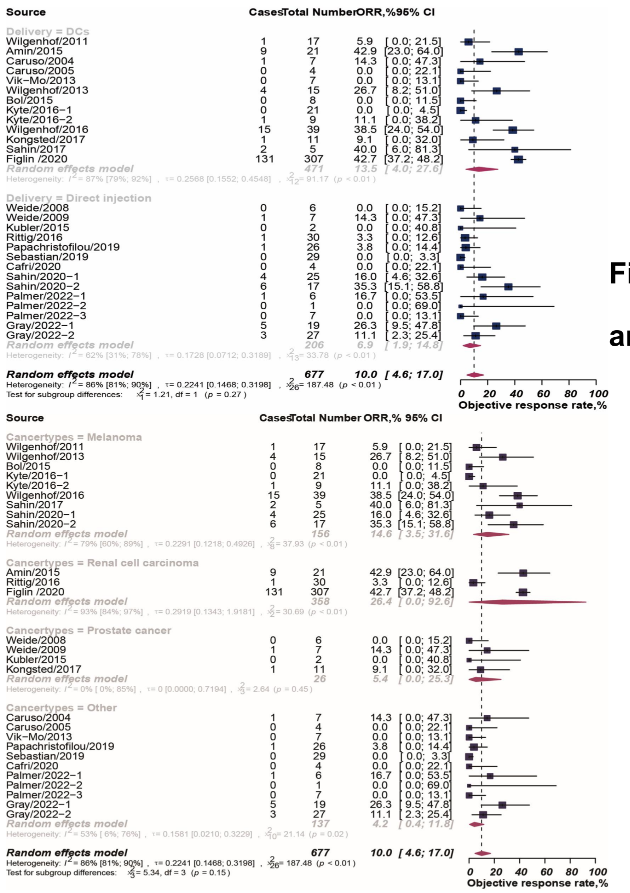

Figure S3. Forest plot of objective response rate (ORR), stratified by cancer types and vehicles

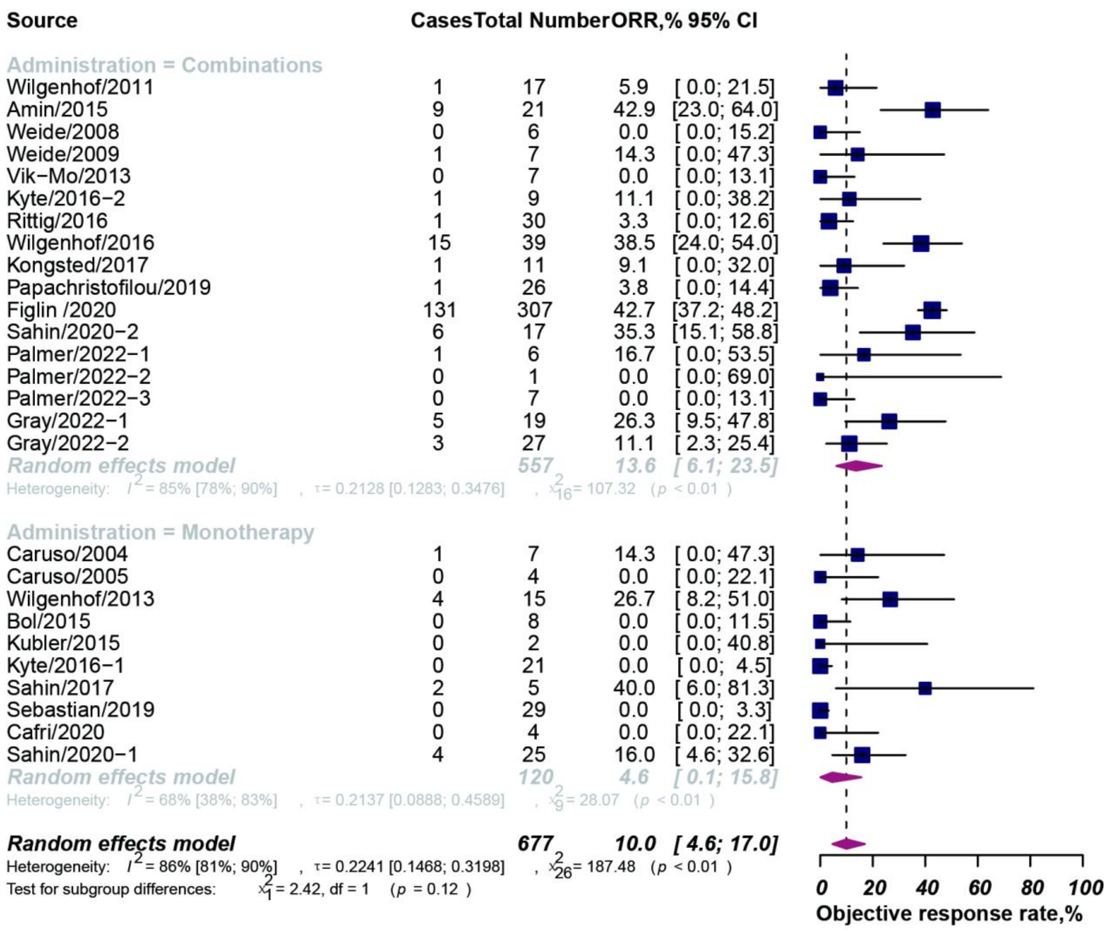

Figure S4. Forest plot of objective response rate (ORR), stratified by treatment modalities

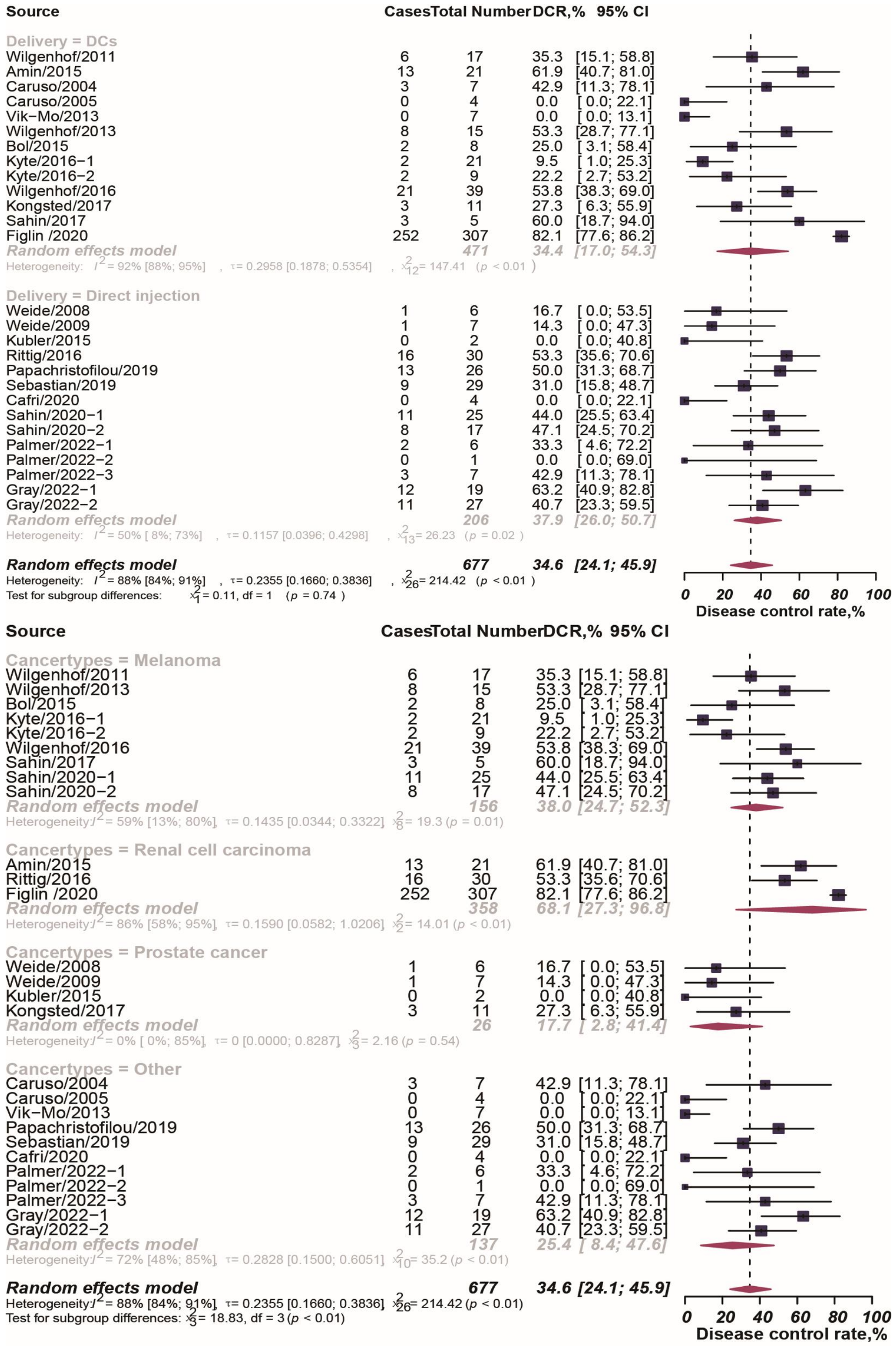

Figure S5.Forest plot of disease control rate (DCR), stratified by cancer types and vehicles

Figure S6. Forest plot of disease control rate (DCR), stratified by treatment modalities

Source	Cases	Total Number	DCR,%	95% CI
Administration = Combinations				
Wilgenhof/2011	6	17	35.3	[15.1; 58.8]
Amin/2015	13	21	61.9	[40.7; 81.0]
Weide/2008	1	6	16.7	[0.0; 53.5]
Weide/2009	1	7	14.3	[0.0; 47.3]
Vik−Mo/2013	0	7	0.0	[0.0; 13.1]
Kyte/2016−2	2	9	22.2	[2.7; 53.2]
Rittig/2016	16	30	53.3	[35.6; 70.6]
Wilgenhof/2016	21	39	53.8	[38.3; 69.0]
Kongsted/2017	3	11	27.3	[6.3; 55.9]
Papachristofilou/2019	13	26	50.0	[31.3; 68.7]
Figlin /2020	252	307	82.1	[77.6; 86.2]
Sahin/2020−2	8	17	47.1	[24.5; 70.2]
Palmer/2022−1	2	6	33.3	[4.6; 72.2]
Palmer/2022−2	0	1	0.0	[0.0; 69.0]
Palmer/2022−3	3	7	42.9	[11.3; 78.1]
Gray/2022−1	12	19	63.2	[40.9; 82.8]
Gray/2022−2	11	27	40.7	[23.3; 59.5]
Random effects model		*557*	*40.8*	*[27.7; 54.5]*
Heterogeneity: $I^2 = 88\%$ [82%; 92%], $\tau = 0.2202$ [0.1388; 0.3959], $\chi^2_{16} = 128.86$ ($p < 0.01$)				
Administration = Monotherapy				
Caruso/2004	3	7	42.9	[11.3; 78.1]
Caruso/2005	0	4	0.0	[0.0; 22.1]
Wilgenhof/2013	8	15	53.3	[28.7; 77.1]
Bol/2015	2	8	25.0	[3.1; 58.4]
Kubler/2015	0	2	0.0	[0.0; 40.8]
Kyte/2016−1	2	21	9.5	[1.0; 25.3]
Sahin/2017	3	5	60.0	[18.7; 94.0]
Sebastian/2019	9	29	31.0	[15.8; 48.7]
Cafri/2020	0	4	0.0	[0.0; 22.1]
Sahin/2020−1	11	25	44.0	[25.5; 63.4]
Random effects model		*120*	*24.1*	*[8.0; 45.5]*
Heterogeneity: $I^2 = 67\%$ [36%; 83%], $\tau = 0.2360$ [0.1099; 0.5980], $\chi^2_{9} = 27.32$ ($p < 0.01$)				
Random effects model		***677***	***34.6***	***[24.1; 45.9]***
Heterogeneity: $I^2 = 88\%$ [84%; 91%], $\tau = 0.2355$ [0.1660; 0.3836], $\chi^2_{26} = 214.42$ ($p < 0.01$)				
Test for subgroup differences: $\chi^2_1 = 2.24$, df = 1 ($p = 0.13$)				

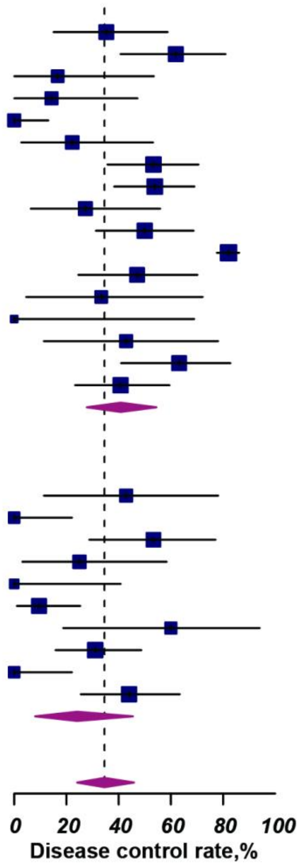

Figure S7.Funnel plot for publication bias regarding 1-year and 2-year progression-free survival (PFS)

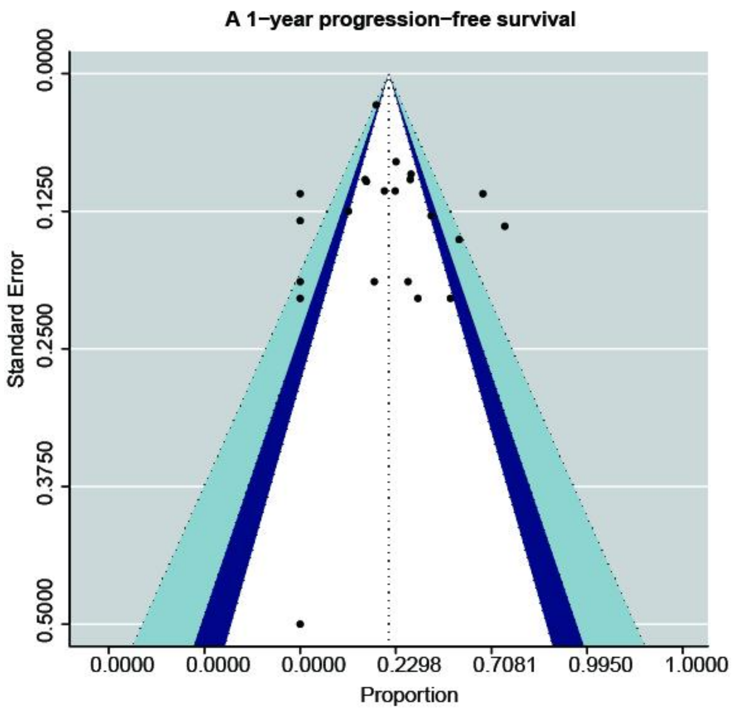

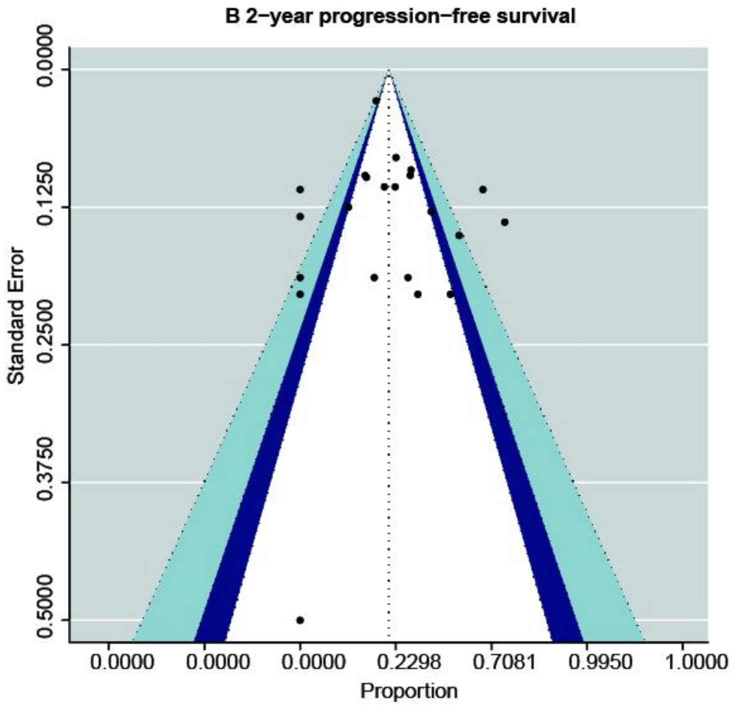

Figure S8. Forest plot of 1-year progression-free survival (1-y PFS), stratified by cancer types and vehicles

Source	Cases	Total Number	1−y PFS,%	95% CI
**Delivery = DCs**				
Wilgenhof/2011	12	35	34.3	[19.7; 50.6]
Amin/2015	10	22	45.5	[25.6; 66.1]
Kongsted/2017	0	21	0.0	[0.0; 4.5]
Caruso/2005	7	7	100.0	[86.9; 100.0]
Vik−Mo/2013	6	7	85.7	[52.7; 100.0]
Wilgenhof/2013	6	15	40.0	[17.5; 65.0]
Kyte/2016	8	22	36.4	[18.0; 57.1]
Wilgenhof/2016	13	39	33.3	[19.6; 48.7]
Sahin/2017	11	13	84.6	[61.0; 98.3]
Mitchell /2015−1	1	6	16.7	[0.0; 53.5]
Mitchell/2015−2	4	6	66.7	[27.8; 95.4]
Batich/2017	8	11	72.7	[44.1; 93.7]
Boudewijns/2020−1	5	27	18.5	[6.4; 35.0]
Boudewijns/2020−2	8	27	29.6	[14.2; 47.9]
Figlin /2020	89	307	29.0	[24.1; 34.2]
Jansen/2020	15	21	71.4	[50.8; 88.2]
Random effects model		*586*	*46.2*	*[27.6; 65.4]*
Heterogeneity: $I^2 = 87\%$ [80%; 91%], $\tau = 0.3331$ [0.2315; 0.5570], $\chi^2_{15} = 113.76$ ($p < 0.01$)				
**Delivery = Direct injection**				
Rittig/2016−1	3	14	21.4	[4.7; 45.8]
Rittig/2016−2	3	16	18.8	[4.1; 40.8]
Papachristofilou/2019	4	26	15.4	[4.4; 31.4]
Sebastian/2019	7	46	15.2	[6.4; 26.9]
Palmer/2022−1	2	6	33.3	[4.6; 72.2]
Palmer/2022−2	0	1	0.0	[0.0; 69.0]
Palmer/2022−3	3	7	42.9	[11.3; 78.1]
Random effects model		*116*	*18.5*	*[11.7; 26.3]*
Heterogeneity: $I^2 = 0\%$ [0%; 71%], $\tau = 0$ [0.0000; 0.3335], $\chi^2_6 = 4.13$ ($p = 0.66$)				
Random effects model		***702***	***38.4***	***[24.8; 53.0]***
Heterogeneity: $I^2 = 83\%$ [76%; 88%], $\tau = 0.2979$ [0.2162; 0.4704], $\chi^2_{22} = 130.01$ ($p < 0.01$)				
Test for subgroup differences: $\chi^2_1 = 9.41$, df = 1 ($p < 0.01$)				

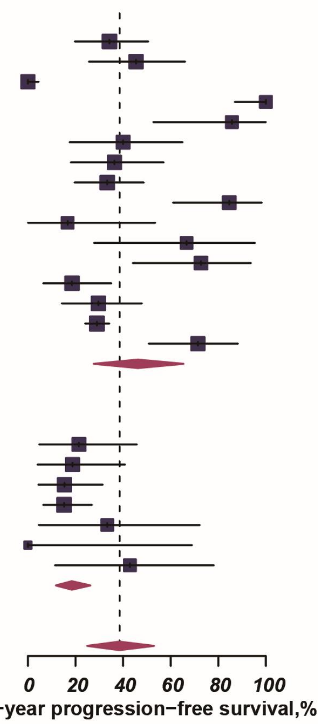

Source	Cases	Total Number	1−y PFS,%	95% CI
**Cancertypes = Melanoma**				
Wilgenhof/2011	12	35	34.3	[19.7; 50.6]
Wilgenhof/2013	6	15	40.0	[17.5; 65.0]
Kyte/2016	8	22	36.4	[18.0; 57.1]
Wilgenhof/2016	13	39	33.3	[19.6; 48.7]
Sahin/2017	11	13	84.6	[61.0; 98.3]
Mitchell /2015−1	1	6	16.7	[0.0; 53.5]
Mitchell/2015−2	4	6	66.7	[27.8; 95.4]
Batich/2017	8	11	72.7	[44.1; 93.7]
Boudewijns/2020−1	5	27	18.5	[6.4; 35.0]
Boudewijns/2020−2	8	27	29.6	[14.2; 47.9]
Jansen/2020	15	21	71.4	[50.8; 88.2]
Random effects model		*222*	*44.7*	*[28.9; 61.0]*
Heterogeneity: $I^2 = 74\%$ [52%; 86%], $\tau = 0.2047$ [0.1129; 0.4183], $\chi^2_{10} = 38.07$ ($p < 0.01$)				
**Cancertypes = Renal cell carcinoma**				
Amin/2015	10	22	45.5	[25.6; 66.1]
Rittig/2016−1	3	14	21.4	[4.7; 45.8]
Rittig/2016−2	3	16	18.8	[4.1; 40.8]
Figlin /2020	89	307	29.0	[24.1; 34.2]
Random effects model		*359*	*29.1*	*[20.8; 38.2]*
Heterogeneity: $I^2 = 24\%$ [0%; 88%], $\tau = 0.0009$ [0.0000; 0.4734], $\chi^2_3 = 3.94$ ($p = 0.27$)				
**Cancertypes = Prostate cancer**				
Kongsted/2017	0	21	0.0	[0.0; 4.5]
**Cancertypes = Other**				
Caruso/2005	7	7	100.0	[86.9; 100.0]
Vik−Mo/2013	6	7	85.7	[52.7; 100.0]
Papachristofilou/2019	4	26	15.4	[4.4; 31.4]
Sebastian/2019	7	46	15.2	[6.4; 26.9]
Palmer/2022−1	2	6	33.3	[4.6; 72.2]
Palmer/2022−2	0	1	0.0	[0.0; 69.0]
Palmer/2022−3	3	7	42.9	[11.3; 78.1]
Random effects model		*100*	*45.0*	*[7.3; 86.7]*
Heterogeneity: $I^2 = 88\%$ [77%; 93%], $\tau = 0.4450$ [0.2467; 1.1214], $\chi^2_6 = 48.67$ ($p < 0.01$)				
Random effects model		***702***	***38.4***	***[24.8; 53.0]***
Heterogeneity: $I^2 = 83\%$ [76%; 88%], $\tau = 0.2979$ [0.2162; 0.4704], $\chi^2_{22} = 130.01$ ($p < 0.01$)				
Test for subgroup differences: $\chi^2_3 = 32.77$, df = 3 ($p < 0.01$)				

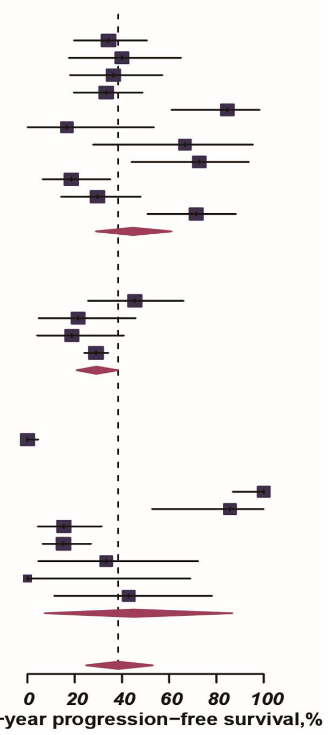

Source	Cases	Total Number	1−y PFS,%	95% CI
Administration = Combinations				
Wilgenhof/2011	12	35	34.3	[19.7; 50.6]
Amin/2015	10	22	45.5	[25.6; 66.1]
Kongsted/2017	0	21	0.0	[0.0; 4.5]
Vik−Mo/2013	6	7	85.7	[52.7; 100.0]
Rittig/2016−1	3	14	21.4	[4.7; 45.8]
Rittig/2016−2	3	16	18.8	[4.1; 40.8]
Wilgenhof/2016	13	39	33.3	[19.6; 48.7]
Papachristofilou/2019	4	26	15.4	[4.4; 31.4]
Mitchell /2015−1	1	6	16.7	[0.0; 53.5]
Mitchell/2015−2	4	6	66.7	[27.8; 95.4]
Batich/2017	8	11	72.7	[44.1; 93.7]
Boudewijns/2020−2	8	27	29.6	[14.2; 47.9]
Figlin /2020	89	307	29.0	[24.1; 34.2]
Palmer/2022−1	2	6	33.3	[4.6; 72.2]
Palmer/2022−2	0	1	0.0	[0.0; 69.0]
Palmer/2022−3	3	7	42.9	[11.3; 78.1]
Random effects model		*551*	*31.5*	*[18.5; 46.2]*
Heterogeneity: I^2 = 75% [60%; 85%], τ = 0.2350 [0.1475; 0.4320], χ^2_{15} = 60.4 (p < 0.01)				
Administration = Monotherapy				
Caruso/2005	7	7	100.0	[86.9; 100.0]
Wilgenhof/2013	6	15	40.0	[17.5; 65.0]
Kyte/2016	8	22	36.4	[18.0; 57.1]
Sahin/2017	11	13	84.6	[61.0; 98.3]
Sebastian/2019	7	46	15.2	[6.4; 26.9]
Boudewijns/2020−1	5	27	18.5	[6.4; 35.0]
Jansen/2020	15	21	71.4	[50.8; 88.2]
Random effects model		*151*	*54.2*	*[18.4; 87.7]*
Heterogeneity: I^2 = 91% [83%; 95%], τ = 0.3890 [0.2312; 0.9218], χ^2_{6} = 63.98 (p < 0.01)				
Random effects model		***702***	***38.4***	***[24.8; 53.0]***
Heterogeneity: I^2 = 83% [76%; 88%], τ = 0.2979 [0.2162; 0.4704], χ^2_{22} = 130.01 (p < 0.01)				
Test for subgroup differences: χ^2_1 = 1.81, df = 1 (p = 0.18)				

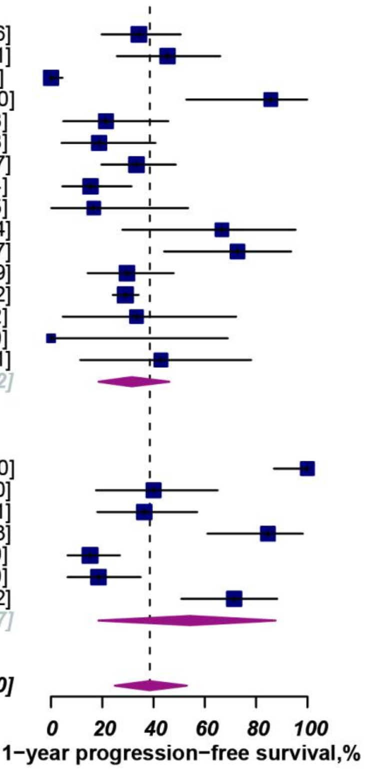

Figure S9. Forest plot of 1-year progression-free survival (1-y PFS), stratified by treatment modalities

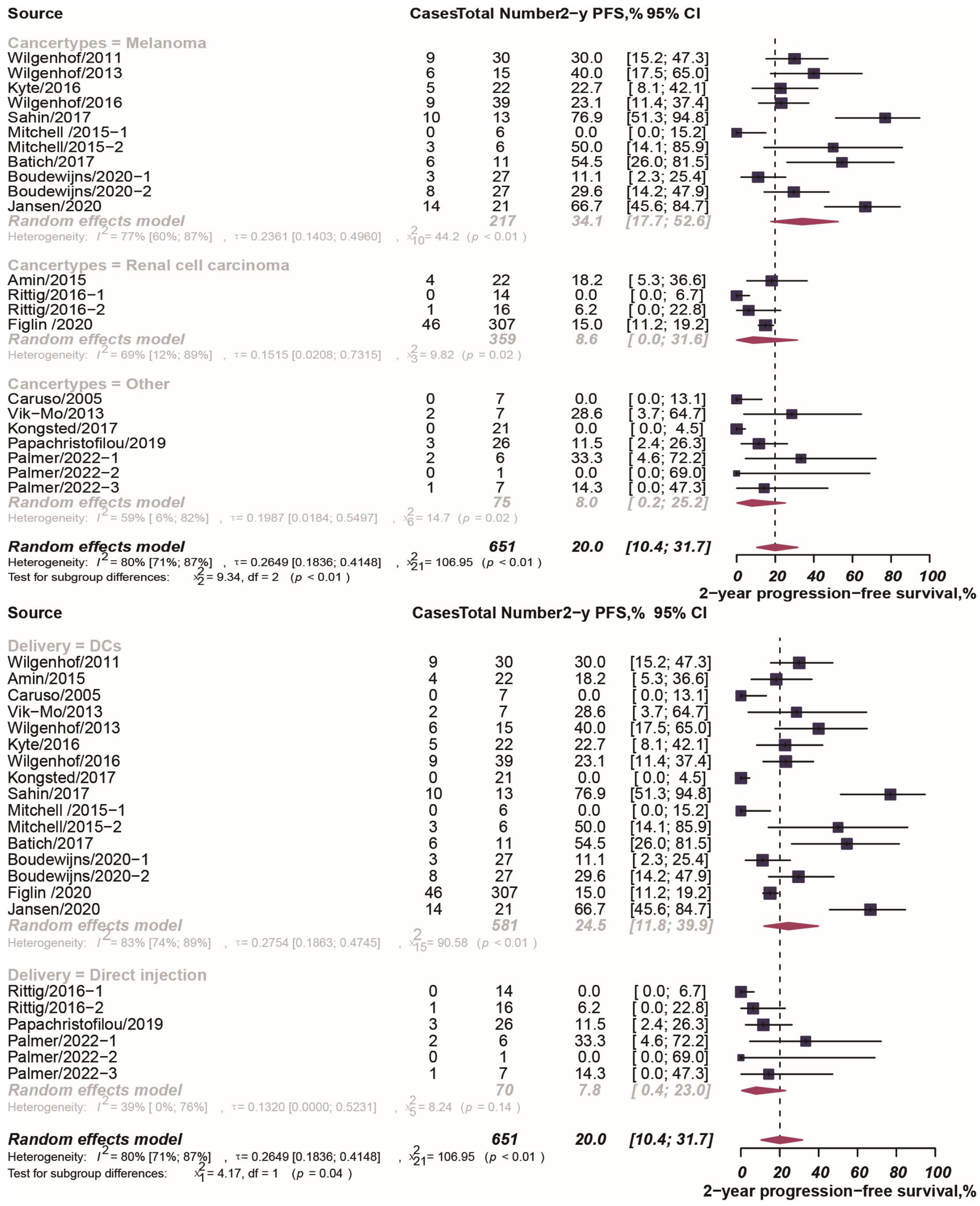

Figure S10.Forest plot of 2-year progression-free survival (2-y PFS), stratified by cancer types and vehicles

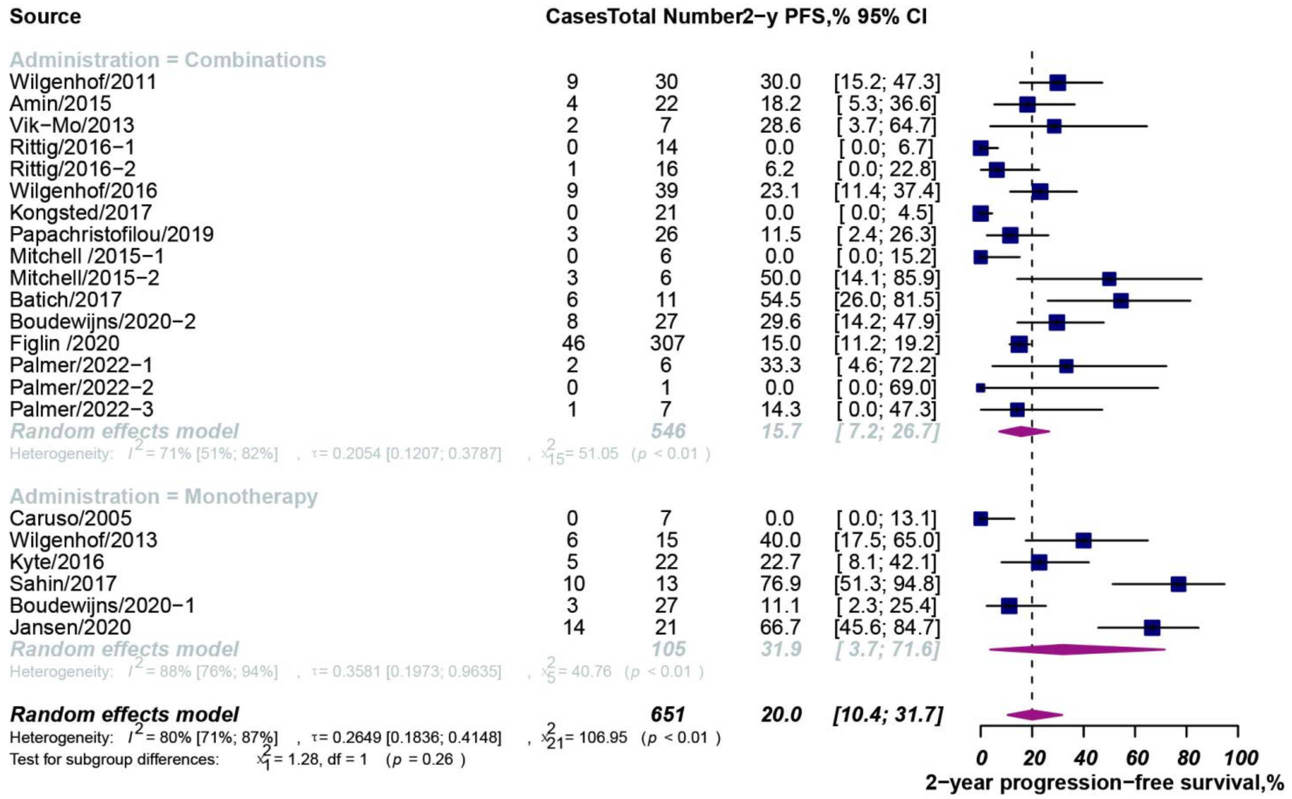

Figure S11.Forest plot of 2-year progression-free survival (2-y PFS), stratified by treatment modalities

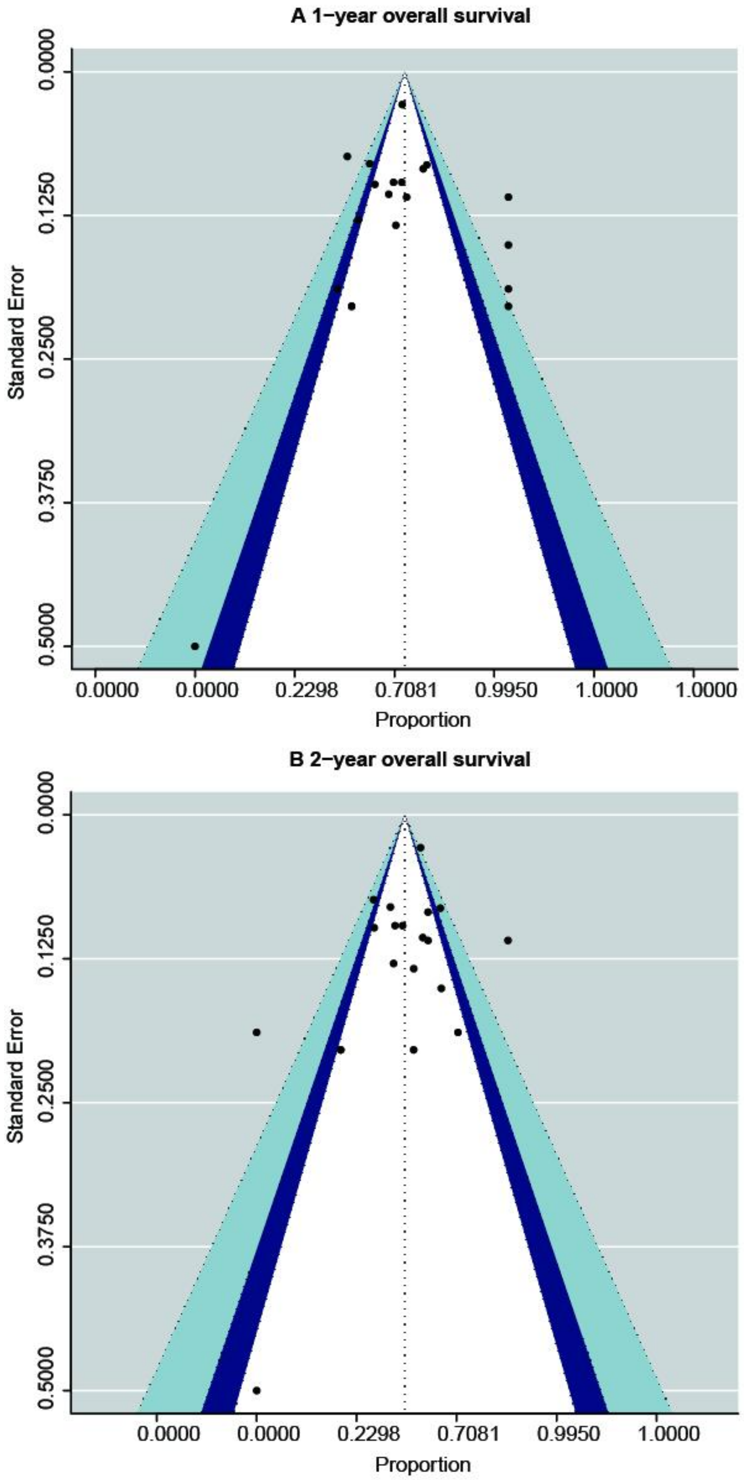

Figure S12. Funnel plot for publication bias regarding 1-year and 2-year overall survival (OS)

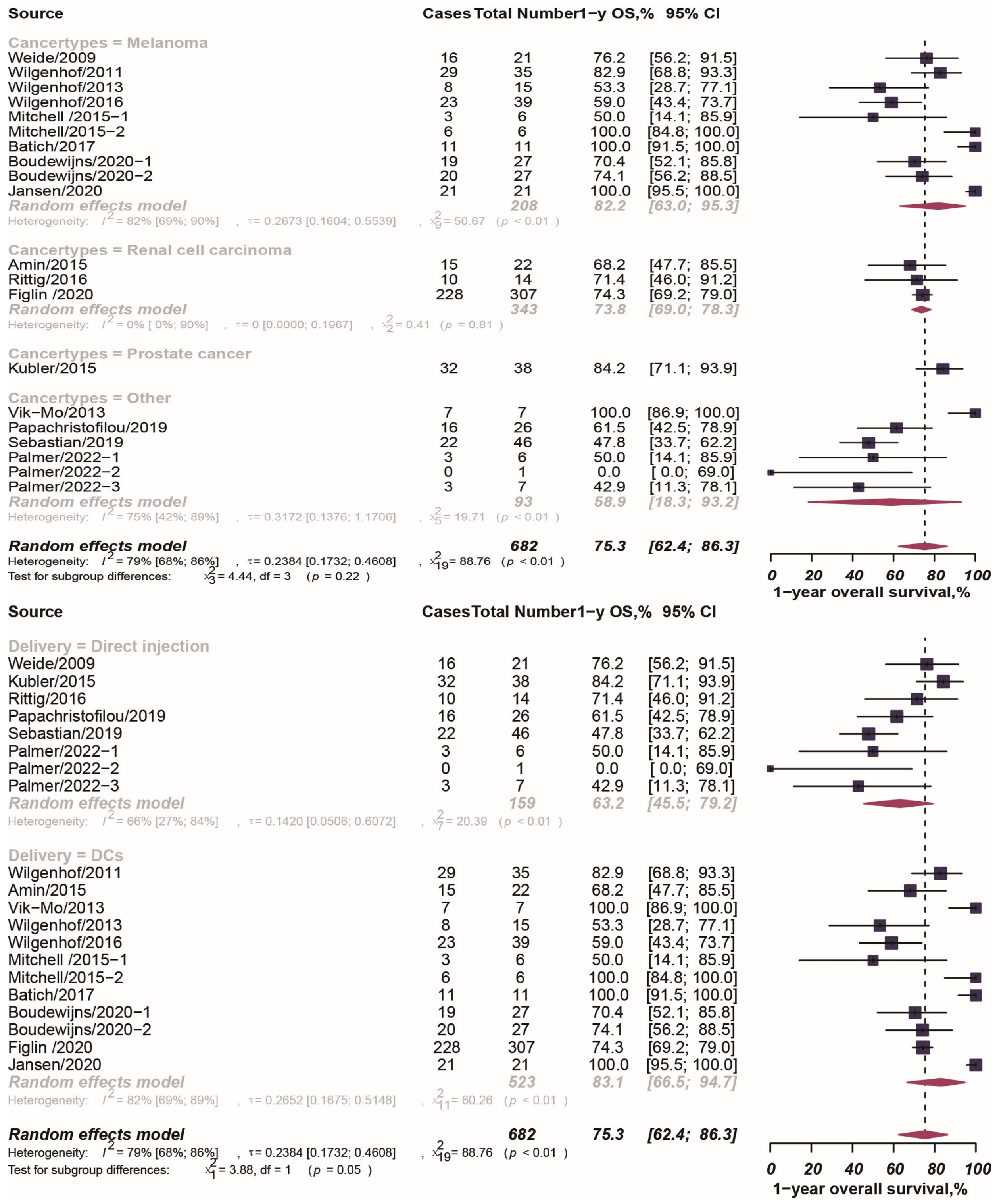

Figure S13. Forest plot of 1-year overall survival (1-y OS), stratified by cancer types and vehicles

Figure S14.Forest plot of 1-year overall survival (1-y OS), stratified by treatment modalities

Source	Cases	Total Number	1−y OS,%	95% CI
Administration = Combinations				
Weide/2009	16	21	76.2	[56.2; 91.5]
Wilgenhof/2011	29	35	82.9	[68.8; 93.3]
Amin/2015	15	22	68.2	[47.7; 85.5]
Vik−Mo/2013	7	7	100.0	[86.9; 100.0]
Rittig/2016	10	14	71.4	[46.0; 91.2]
Wilgenhof/2016	23	39	59.0	[43.4; 73.7]
Papachristofilou/2019	16	26	61.5	[42.5; 78.9]
Mitchell /2015−1	3	6	50.0	[14.1; 85.9]
Mitchell/2015−2	6	6	100.0	[84.8; 100.0]
Batich/2017	11	11	100.0	[91.5; 100.0]
Boudewijns/2020−2	20	27	74.1	[56.2; 88.5]
Figlin /2020	228	307	74.3	[69.2; 79.0]
Palmer/2022−1	3	6	50.0	[14.1; 85.9]
Palmer/2022−2	0	1	0.0	[0.0; 69.0]
Palmer/2022−3	3	7	42.9	[11.3; 78.1]
Random effects model		*535*	*75.1*	*[59.8; 87.7]*
Heterogeneity: $I^2 = 69\%$ [48%; 82%], $\tau = 0.2129$ [0.1441; 0.5332], $\chi^2_{14} = 45.66$ ($p < 0.01$)				
Administration = Monotherapy				
Kubler/2015	32	38	84.2	[71.1; 93.9]
Wilgenhof/2013	8	15	53.3	[28.7; 77.1]
Sebastian/2019	22	46	47.8	[33.7; 62.2]
Boudewijns/2020−1	19	27	70.4	[52.1; 85.8]
Jansen/2020	21	21	100.0	[95.5; 100.0]
Random effects model		*147*	*76.2*	*[37.6; 98.8]*
Heterogeneity: $I^2 = 91\%$ [81%; 95%], $\tau = 0.3057$ [0.1646; 0.9269], $\chi^2_4 = 43.08$ ($p < 0.01$)				
Random effects model		***682***	***75.3***	***[62.4; 86.3]***
Heterogeneity: $I^2 = 79\%$ [68%; 86%], $\tau = 0.2384$ [0.1732; 0.4608], $\chi^2_{19} = 88.76$ ($p < 0.01$)				
Test for subgroup differences: $\chi^2_1 = 0.01$, df = 1 ($p = 0.94$)				

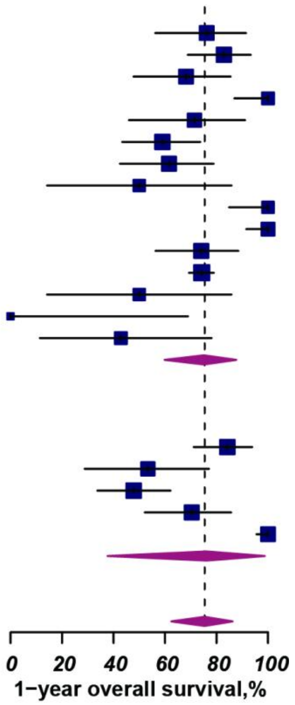

Figure S15. Forest plot of 2-year overall survival (2-y OS), stratified by cancer types and vehicles

Source	Cases	Total Number	2−year OS,%	95% CI
Cancertypes = Melanoma				
Weide/2009	12	21	57.1	[36.0; 77.0]
Wilgenhof/2011	20	35	57.1	[40.7; 72.9]
Wilgenhof/2013	6	15	40.0	[17.5; 65.0]
Wilgenhof/2016	15	39	38.5	[24.0; 54.0]
Mitchell /2015−1	1	6	16.7	[0.0; 53.5]
Mitchell/2015−2	3	6	50.0	[14.1; 85.9]
Batich/2017	7	11	63.6	[34.5; 88.1]
Boudewijns/2020−1	11	27	40.7	[23.3; 59.5]
Boudewijns/2020−2	12	27	44.4	[26.5; 63.1]
Jansen/2020	19	21	90.5	[74.7; 99.0]
Common effect model		*208*	*51.5*	*[44.7; 58.3]*
Random effects model			*51.5*	*[36.8; 66.1]*
Heterogeneity: I^2 = 67% [37%; 83%], τ = 0.1653 [0.0754; 0.3674], χ^2_9 = 27.67 (p < 0.01)				
Cancertypes = Renal cell carcinoma				
Amin/2015	12	22	54.5	[33.9; 74.4]
Rittig/2016	7	14	50.0	[25.0; 75.0]
Figlin /2020	164	307	53.4	[47.8; 59.0]
Common effect model		*343*	*53.4*	*[48.1; 58.6]*
Random effects model			*53.4*	*[51.1; 55.6]*
Heterogeneity: I^2 = 0% [0%; 90%], τ = 0 [0.0000; 0.0858], χ^2_2 = 0.08 (p = 0.96)				
Cancertypes = Prostate cancer				
Kubler/2015	24	38	63.2	[47.4; 77.6]
Cancertypes = Other				
Vik−Mo/2013	5	7	71.4	[35.3; 96.3]
Papachristofilou/2019	8	26	30.8	[14.9; 49.5]
Sebastian/2019	14	46	30.4	[18.1; 44.4]
Palmer/2022−1	1	6	16.7	[0.0; 53.5]
Palmer/2022−2	0	1	0.0	[0.0; 69.0]
Palmer/2022−3	0	7	0.0	[0.0; 13.1]
Common effect model		*93*	*27.9*	*[19.3; 37.4]*
Random effects model			*22.7*	*[1.6; 58.3]*
Heterogeneity: I^2 = 69% [28%; 87%], τ = 0.2867 [0.0992; 0.9073], χ^2_5 = 16.28 (p < 0.01)				
Common effect model		***682***	***49.8***	***[46.0; 53.5]***
Random effects model			***45.5***	***[34.0; 57.2]***
Heterogeneity: I^2 = 72% [56%; 82%], τ = 0.1911 [0.1300; 0.3736], χ^2_{19} = 67.96 (p < 0.01)				
Test for subgroup differences (common effect): χ^2_3 = 23.94, df = 3 (p < 0.01)				
Test for subgroup differences (random effects): χ^2_3 = 6.55, df = 3 (p = 0.09)				

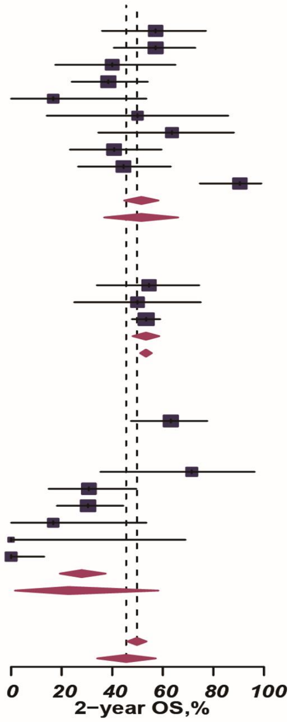

Source	Cases	Total Number	2−year OS,%	95% CI
Delivery = Direct injection				
Weide/2009	12	21	57.1	[36.0; 77.0]
Kubler/2015	24	38	63.2	[47.4; 77.6]
Rittig/2016	7	14	50.0	[25.0; 75.0]
Papachristofilou/2019	8	26	30.8	[14.9; 49.5]
Sebastian/2019	14	46	30.4	[18.1; 44.4]
Palmer/2022−1	1	6	16.7	[0.0; 53.5]
Palmer/2022−2	0	1	0.0	[0.0; 69.0]
Palmer/2022−3	0	7	0.0	[0.0; 13.1]
Common effect model		*159*	*39.8*	*[32.4; 47.5]*
Random effects model			*31.9*	*[10.9; 57.8]*
Heterogeneity: I^2 = 77% [55%; 89%], τ = 0.2575 [0.1292; 0.6764], χ^2_7 = 30.87 (p < 0.01)				
Delivery = DCs				
Wilgenhof/2011	20	35	57.1	[40.7; 72.9]
Amin/2015	12	22	54.5	[33.9; 74.4]
Vik−Mo/2013	5	7	71.4	[35.3; 96.3]
Wilgenhof/2013	6	15	40.0	[17.5; 65.0]
Wilgenhof/2016	15	39	38.5	[24.0; 54.0]
Mitchell /2015−1	1	6	16.7	[0.0; 53.5]
Mitchell/2015−2	3	6	50.0	[14.1; 85.9]
Batich/2017	7	11	63.6	[34.5; 88.1]
Boudewijns/2020−1	11	27	40.7	[23.3; 59.5]
Boudewijns/2020−2	12	27	44.4	[26.5; 63.1]
Figlin /2020	164	307	53.4	[47.8; 59.0]
Jansen/2020	19	21	90.5	[74.7; 99.0]
Common effect model		*523*	*52.8*	*[48.5; 57.1]*
Random effects model			*52.7*	*[40.7; 64.5]*
Heterogeneity: I^2 = 62% [28%; 80%], τ = 0.1417 [0.0626; 0.3146], χ^2_{11} = 28.77 (p < 0.01)				
Common effect model		***682***	***49.8***	***[46.0; 53.5]***
Random effects model			***45.5***	***[34.0; 57.2]***
Heterogeneity: I^2 = 72% [56%; 82%], τ = 0.1911 [0.1300; 0.3736], χ^2_{19} = 67.96 (p < 0.01)				
Test for subgroup differences (common effect): χ^2_1 = 8.32, df = 1 (p < 0.01)				
Test for subgroup differences (random effects): χ^2_1 = 2.91, df = 1 (p = 0.09)				

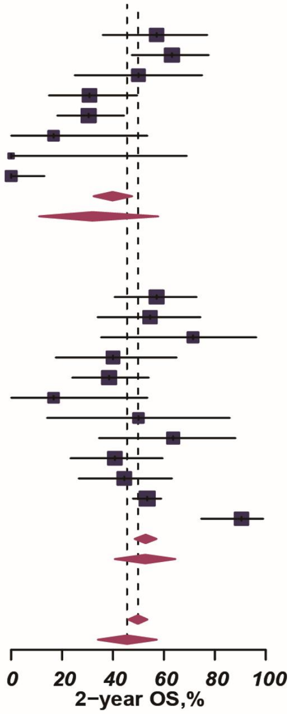

Source	Cases	Total Number	2−year OS,%	95% CI
Administration = Combinations				
Weide/2009	12	21	57.1	[36.0; 77.0]
Wilgenhof/2011	20	35	57.1	[40.7; 72.9]
Amin/2015	12	22	54.5	[33.9; 74.4]
Vik−Mo/2013	5	7	71.4	[35.3; 96.3]
Rittig/2016	7	14	50.0	[25.0; 75.0]
Wilgenhof/2016	15	39	38.5	[24.0; 54.0]
Papachristofilou/2019	8	26	30.8	[14.9; 49.5]
Mitchell /2015−1	1	6	16.7	[0.0; 53.5]
Mitchell/2015−2	3	6	50.0	[14.1; 85.9]
Batich/2017	7	11	63.6	[34.5; 88.1]
Boudewijns/2020−2	12	27	44.4	[26.5; 63.1]
Figlin /2020	164	307	53.4	[47.8; 59.0]
Palmer/2022−1	1	6	16.7	[0.0; 53.5]
Palmer/2022−2	0	1	0.0	[0.0; 69.0]
Palmer/2022−3	0	7	0.0	[0.0; 13.1]
Common effect model		*535*	*49.4*	*[45.2; 53.6]*
Random effects model			*42.9*	*[30.4; 56.0]*
Heterogeneity: $I^2 = 63\%$ [35%; 79%], $\tau = 0.1534$ [0.0885; 0.4097], $\chi^2_{14} = 37.54$ ($p < 0.01$)				
Administration = Monotherapy				
Kubler/2015	24	38	63.2	[47.4; 77.6]
Wilgenhof/2013	6	15	40.0	[17.5; 65.0]
Sebastian/2019	14	46	30.4	[18.1; 44.4]
Boudewijns/2020−1	11	27	40.7	[23.3; 59.5]
Jansen/2020	19	21	90.5	[74.7; 99.0]
Common effect model		*147*	*51.2*	*[43.1; 59.2]*
Random effects model			*53.9*	*[22.3; 83.9]*
Heterogeneity: $I^2 = 87\%$ [71%; 94%], $\tau = 0.2482$ [0.1259; 0.7677], $\chi^2_{4} = 30.28$ ($p < 0.01$)				
Common effect model		***682***	***49.8***	***[46.0; 53.5]***
Random effects model			***45.5***	***[34.0; 57.2]***

Heterogeneity: $I^2 = 72\%$ [56%; 82%], $\tau = 0.1911$ [0.1300; 0.3736], $\chi^2_{19} = 67.96$ ($p < 0.01$)
Test for subgroup differences (common effect): $\chi^2_1 = 0.14$, df = 1 ($p = 0.71$)
Test for subgroup differences (random effects): $\chi^2_1 = 0.67$, df = 1 ($p = 0.41$)

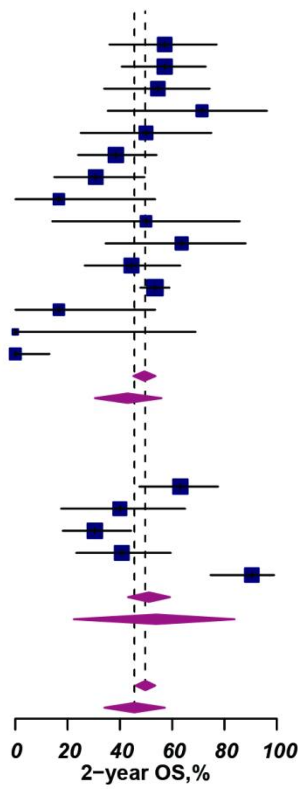

Figure S16.Forest plot of 2-year overall survival (2-y OS), stratified by treatment modalities

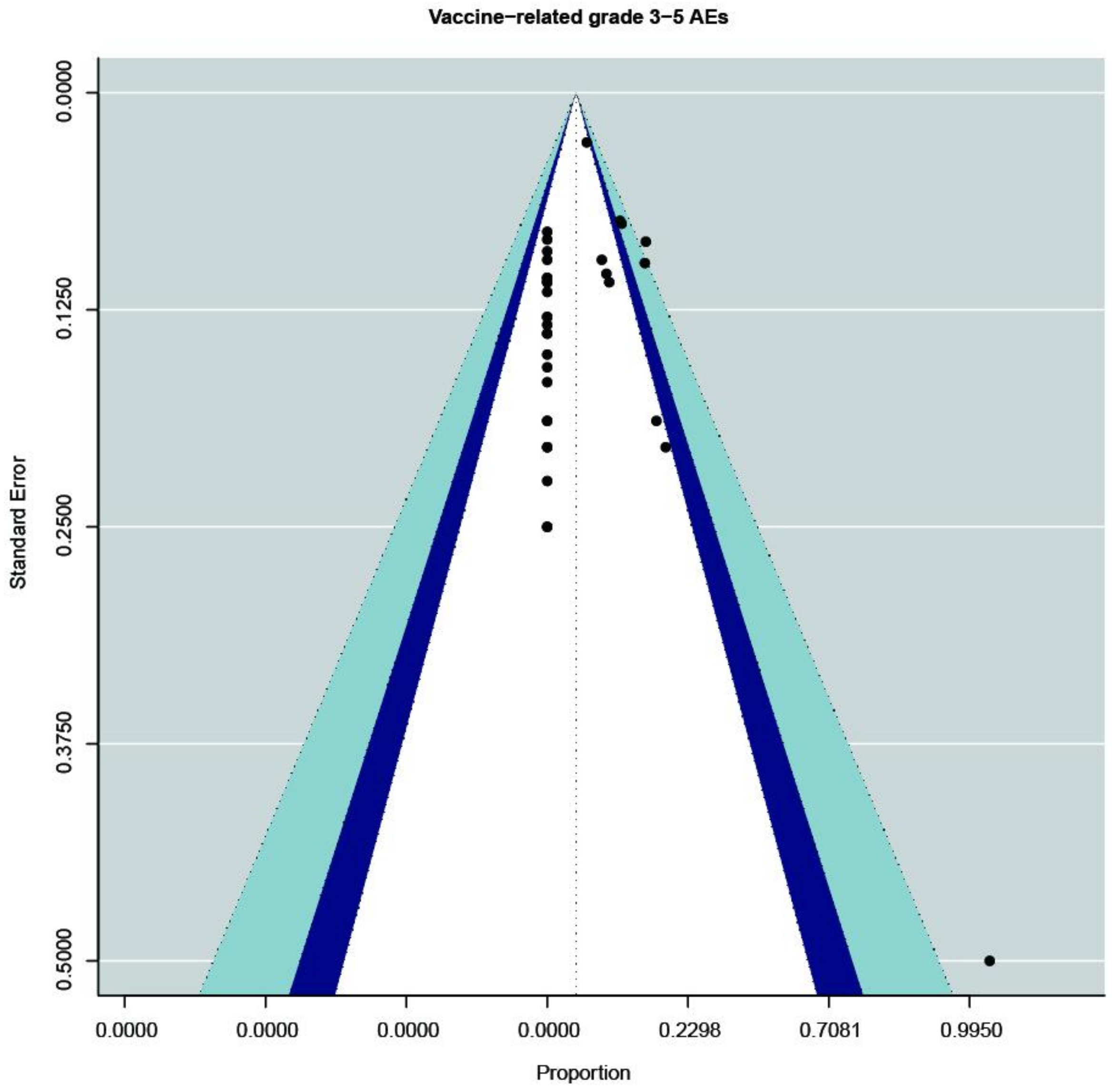

Figure S17. Funnel plot for publication bias regarding vaccine-related grade 3-5 AEs

Study	Cancer types	Delivery	Administration	Patients	ORR,%	95% CI
Wilgenhof/2011	Melanoma	DCs	Combinations	17	5.9	[0.0; 21.5]
Wilgenhof/2013	Melanoma	DCs	Monotherapy	15	26.7	[8.2; 51.0]
Amin/2015	Renal cell carcinoma	DCs	Combinations	21	42.9	[23.0; 64.0]
Kyte/2016−1	Melanoma	DCs	Monotherapy	21	0.0	[0.0; 4.5]
Rittig/2016	Renal cell carcinoma	Direct injection	Combinations	30	3.3	[0.0; 12.6]
Wilgenhof/2016	Melanoma	DCs	Combinations	39	38.5	[24.0; 54.0]
Kongsted/2017	Prostate cancer	DCs	Combinations	11	9.1	[0.0; 32.0]
Papachristofilou/2019	NSCLC	Direct injection	Combinations	26	3.8	[0.0; 14.4]
Sebastian/2019	NSCLC	Direct injection	Monotherapy	29	0.0	[0.0; 3.3]
Figlin /2020	Renal cell carcinoma	DCs	Combinations	307	42.7	[37.2; 48.2]
Sahin/2020−1	Melanoma	Direct injection	Monotherapy	25	16.0	[4.6; 32.6]
Sahin/2020−2	Melanoma	Direct injection	Combinations	17	35.3	[15.1; 58.8]
Gray/2022−1	NSCLC	Direct injection	Combinations	19	26.3	[9.5; 47.8]
Gray/2022−2	NSCLC	Direct injection	Combinations	27	11.1	[2.3; 25.4]
Common effect model				***604***	***26.5***	***[23.0; 30.1]***
Random effects model					***14.8***	***[6.1; 26.5]***
Prediction interval						***[0.0; 63.9]***

Heterogeneity: $I^2 = 91\%$ [86%; 94%], $\tau = 0.2339$ [0.1537; 0.3894], $\chi^2_{13} = 142.09$ ($p < 0.01$)

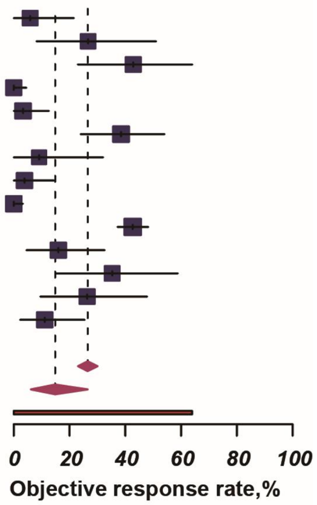

Study	Cancer types	Delivery	Criteria	Patients	DCR,%	95% CI
Wilgenhof/2011	Melanoma	DCs	RECIST	17	35.3	[15.1; 58.8]
Wilgenhof/2013	Melanoma	DCs	RECIST	15	53.3	[28.7; 77.1]
Amin/2015	Renal cell carcinoma	DCs	RECIST	21	61.9	[40.7; 81.0]
Kyte/2016−1	Melanoma	DCs	RECIST	21	9.5	[1.0; 25.3]
Rittig/2016	Renal cell carcinoma	Direct injection	RECIST	30	53.3	[35.6; 70.6]
Wilgenhof/2016	Melanoma	DCs	irRC	39	53.8	[38.3; 69.0]
Kongsted/2017	Prostate cancer	DCs	RECIST	11	27.3	[6.3; 55.9]
Papachristofilou/2019	NSCLC	Direct injection	RECIST	26	50.0	[31.3; 68.7]
Sebastian/2019	NSCLC	Direct injection	RECIST	29	31.0	[15.8; 48.7]
Figlin /2020	Renal cell carcinoma	DCs	RECIST	307	82.1	[77.6; 86.2]
Sahin/2020−1	Melanoma	Direct injection	RECIST	25	44.0	[25.5; 63.4]
Sahin/2020−2	Melanoma	Direct injection	irRECIST	17	47.1	[24.5; 70.2]
Gray/2022−1	NSCLC	Direct injection	RECIST	19	63.2	[40.9; 82.8]
Gray/2022−2	NSCLC	Direct injection	RECIST	27	40.7	[23.3; 59.5]
Common effect model				***604***	***64.6***	***[60.8; 68.4]***
Random effects model					***47.4***	***[36.1; 58.9]***
Prediction interval						***[11.7; 84.7]***

Heterogeneity: $I^2 = 90\%$ [85%; 93%], $\tau = 0.1796$ [0.1106; 0.2978], $\chi^2_{13} = 127.00$ ($p < 0.01$)

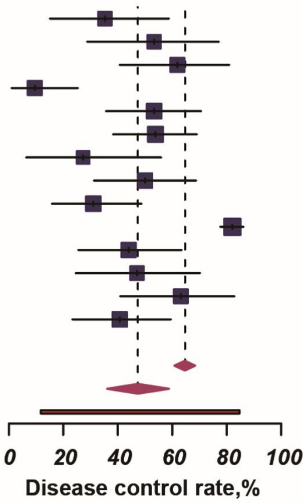

Figure S18.Forest plot of objective response rate (ORR) and disease control rate (DCR), clinical trials with a sample size of less than 10 patients were excluded

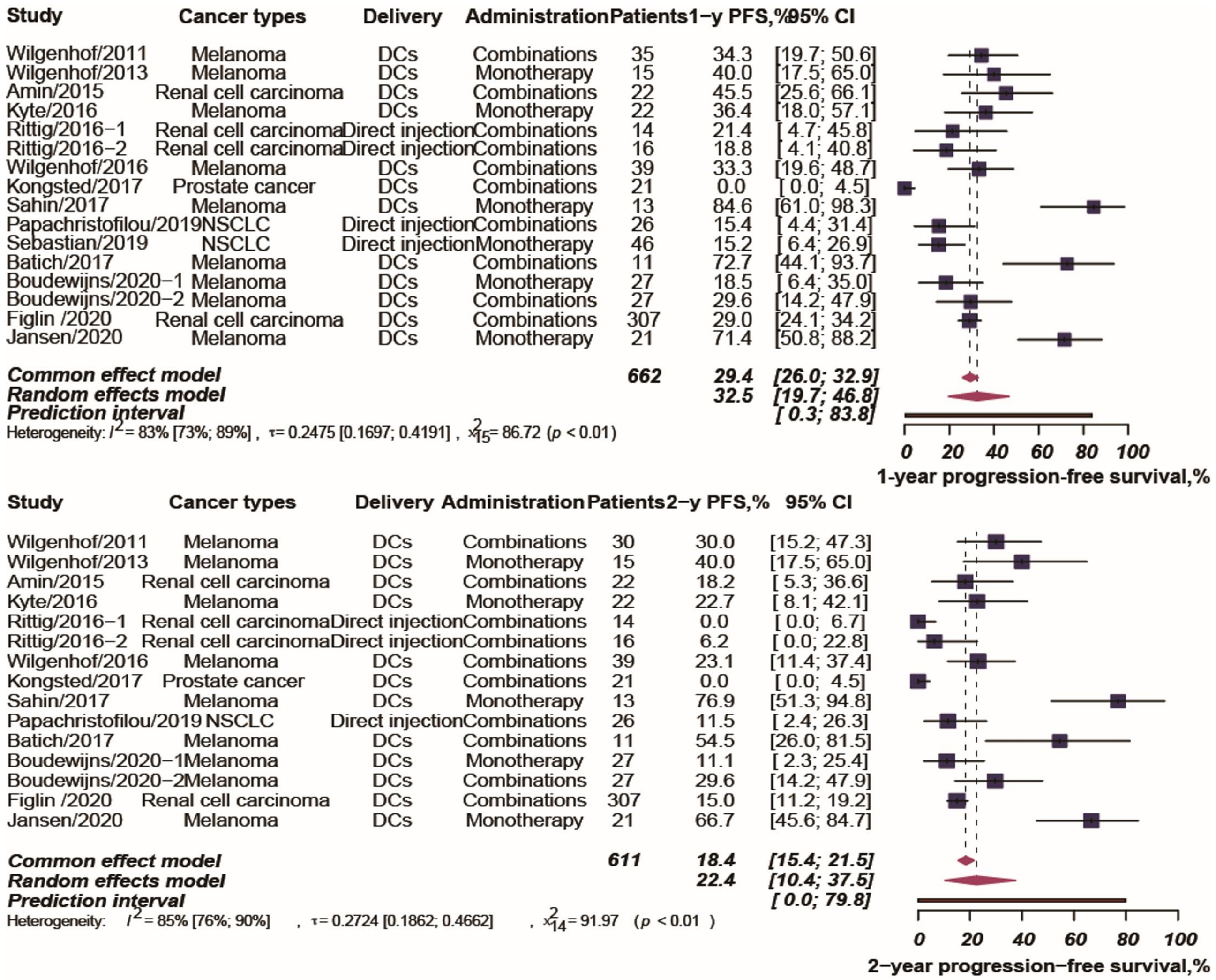

Figure S19. Forest plot of 1-year and 2-year progression-free survival (PFS), clinical trials with a sample size of less than 10 patients were excluded

Study	Cancer types	Delivery	Administration	Patients	1−y OS,%	95% CI
Weide/2009	Melanoma	Direct injection	Combinations	21	76.2	[56.2; 91.5]
Wilgenhof/2011	Melanoma	DCs	Combinations	35	82.9	[68.8; 93.3]
Amin/2015	Renal cell carcinoma	DCs	Combinations	22	68.2	[47.7; 85.5]
Kubler/2015	Prostate cancer	Direct injection	Monotherapy	38	84.2	[71.1; 93.9]
Wilgenhof/2013	Melanoma	DCs	Monotherapy	15	53.3	[28.7; 77.1]
Rittig/2016	Renal cell carcinoma	Direct injection	Combinations	14	71.4	[46.0; 91.2]
Wilgenhof/2016	Melanoma	DCs	Combinations	39	59.0	[43.4; 73.7]
Papachristofilou/2019	NSCLC	Direct injection	Combinations	26	61.5	[42.5; 78.9]
Sebastian/2019	NSCLC	Direct injection	Monotherapy	46	47.8	[33.7; 62.2]
Batich/2017	Melanoma	DCs	Combinations	11	100.0	[91.5; 100.0]
Boudewijns/2020−1	Melanoma	DCs	Monotherapy	27	70.4	[52.1; 85.8]
Boudewijns/2020−2	Melanoma	DCs	Combinations	27	74.1	[56.2; 88.5]
Figlin /2020	Renal cell carcinoma	DCs	Combinations	307	74.3	[69.2; 79.0]
Jansen/2020	Melanoma	DCs	Monotherapy	21	100.0	[95.5; 100.0]
Common effect model				***649***	***73.8***	***[70.4; 77.1]***
Random effects model					***75.8***	***[63.5; 86.3]***
Prediction interval						***[31.7; 99.7]***

Heterogeneity: $I^2 = 80\%$ [66%; 88%], $\tau = 0.2018$ [0.1310; 0.3705], $\chi^2_{13} = 63.67$ ($p < 0.01$)

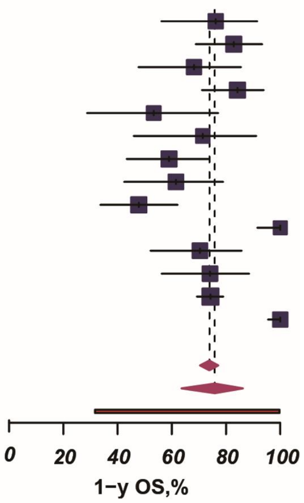

Study	Cancer types	Delivery	Administration	Patients	2−y OS,%	95% CI
Weide/2009	Melanoma	Direct injection	Combinations	21	57.1	[36.0; 77.0]
Wilgenhof/2011	Melanoma	DCs	Combinations	35	57.1	[40.7; 72.9]
Amin/2015	Renal cell carcinoma	DCs	Combinations	22	54.5	[33.9; 74.4]
Kubler/2015	Prostate cancer	Direct injection	Monotherapy	38	63.2	[47.4; 77.6]
Wilgenhof/2013	Melanoma	DCs	Monotherapy	15	40.0	[17.5; 65.0]
Rittig/2016	Renal cell carcinoma	Direct injection	Combinations	14	50.0	[25.0; 75.0]
Wilgenhof/2016	Melanoma	DCs	Combinations	39	38.5	[24.0; 54.0]
Papachristofilou/2019	NSCLC	Direct injection	Combinations	26	30.8	[14.9; 49.5]
Sebastian/2019	NSCLC	Direct injection	Monotherapy	46	30.4	[18.1; 44.4]
Batich/2017	Melanoma	DCs	Combinations	11	63.6	[34.5; 88.1]
Boudewijns/2020−1	Melanoma	DCs	Monotherapy	27	40.7	[23.3; 59.5]
Boudewijns/2020−2	Melanoma	DCs	Combinations	27	44.4	[26.5; 63.1]
Figlin /2020	Renal cell carcinoma	DCs	Combinations	307	53.4	[47.8; 59.0]
Jansen/2020	Melanoma	DCs	Monotherapy	21	90.5	[74.7; 99.0]
Common effect model				***649***	***51.2***	***[47.3; 55.0]***
Random effects model					***50.9***	***[41.1; 60.6]***
Prediction interval						***[21.4; 80.0]***

Heterogeneity: $I^2 = 68\%$ [43%; 81%], $\tau = 0.1364$ [0.0736; 0.2571], $\chi^2_{13} = 40.12$ ($p < 0.01$)

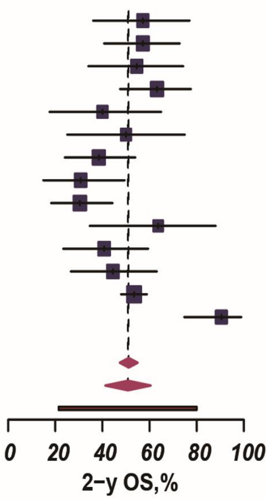

Figure S20. Forest plot of 1-year and 2-year overall survival (2-y OS),clinical trials with a sample size of less than 10 patients were excluded

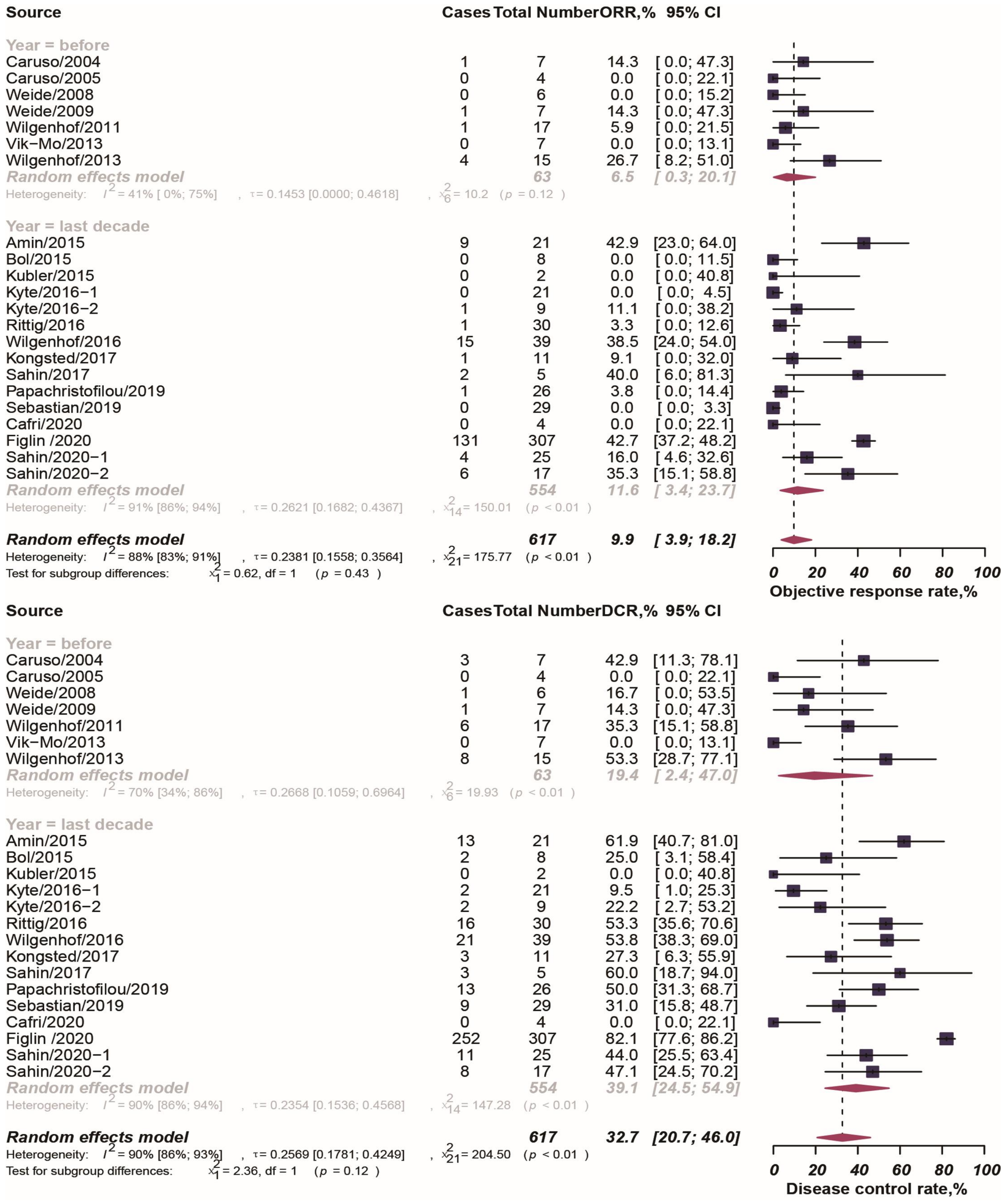

Figure S21. Forest plot of objective response rate (ORR) and disease control rate (DCR), stratified by published year.

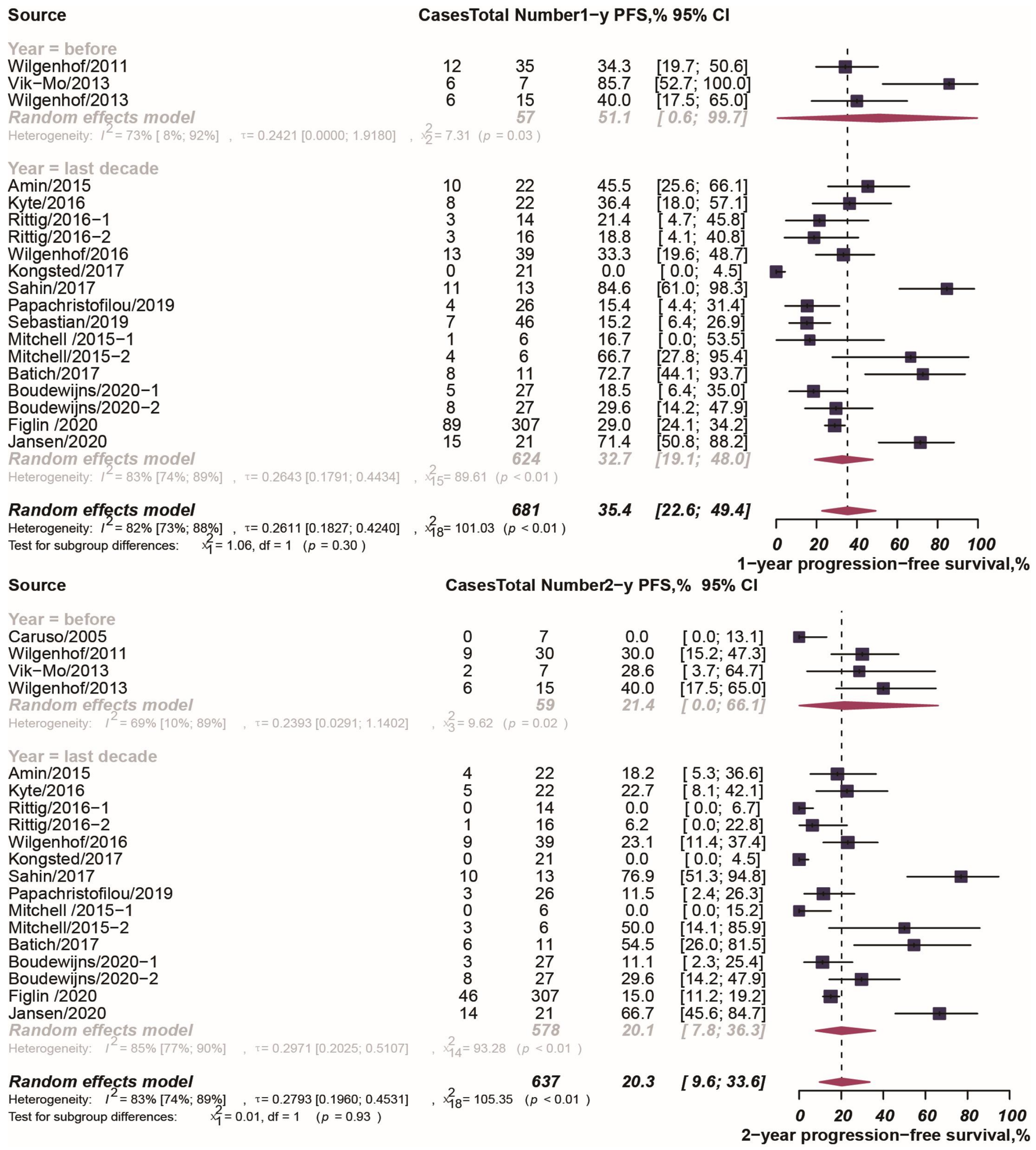

Figure S22. Forest plot of 1-year and 2-year progression-free survival (PFS), stratified by published year

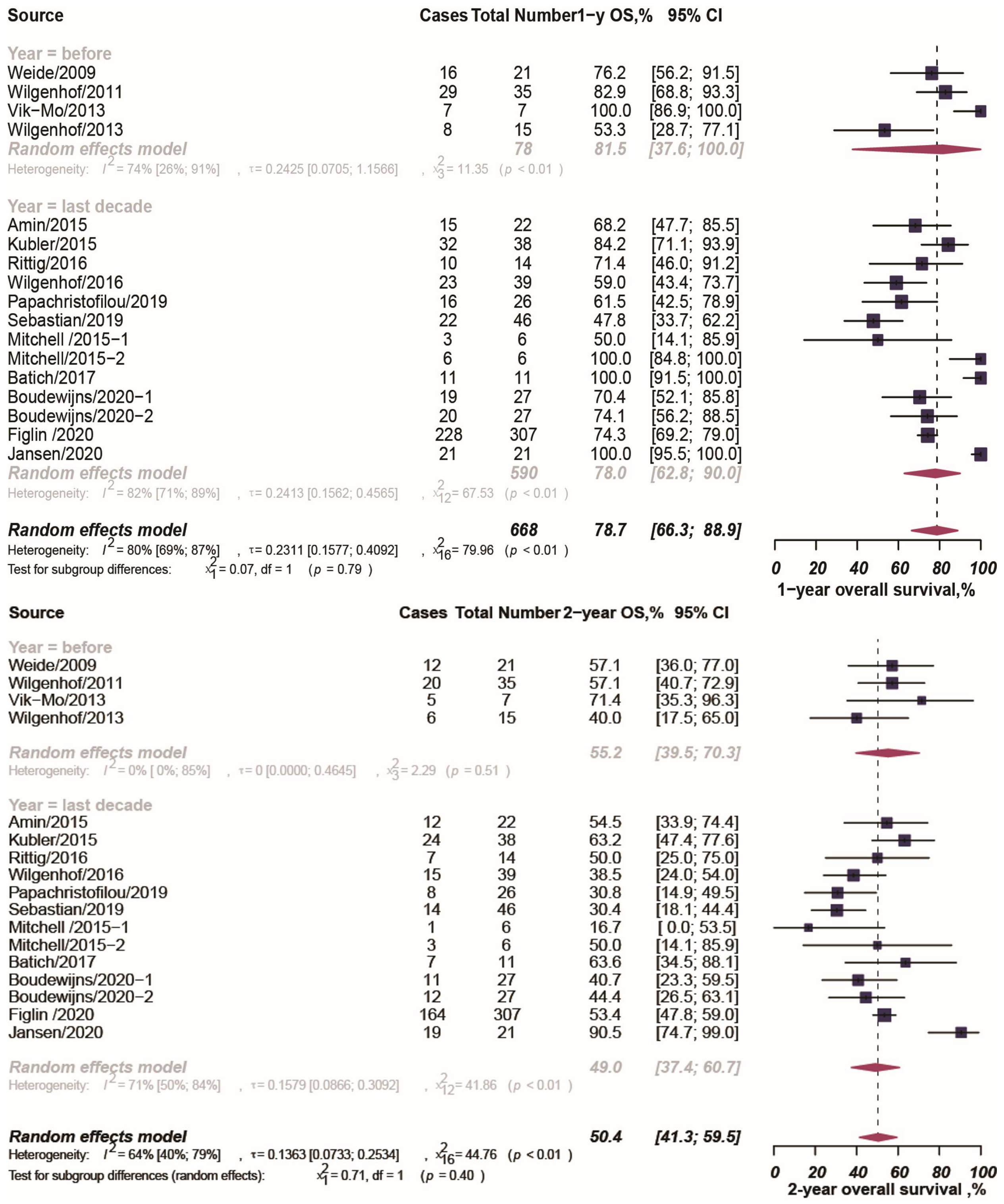

Figure S23. Forest plot of 1-year and 2-yearoverall survival (OS), stratified by published year

Method S1. R Code for subgroup analysis (take ORR for example).

```
library(metafor)
library(meta)

a=read.csv("D:/proportion_meta_data/ORR.csv", header=T, sep=",")
ies.logit=escalc(xi=event,ni=n,measure="PAS",data=a)
pes.logit=rma(yi,vi,data=ies.logit,method="REML",test="knha")
pes.forest=metaprop(event,n,Study,data=a,sm="PAS",method.tau="REML",method.ci ="NAsm",
                    incr=0.5,allincr=FALSE,addincr=FALSE,hakn=TRUE)
pes.forest
pes.forest$event <- round(pes.forest$event)
forest(pes.forest,
       xlim=c(0,100),pscale=100,comb.fixed=FALSE,comb.random=TRUE,
lty.random=2,type.study="square",type.random="diamond",hetlab = "Heterogeneity:",
       rightcols=FALSE,
       leftcols=c("studlab","Cancertypes","Delivery","Criteria","n","effect", "ci"),
       leftlabs=c("Study","Cancer types","Delivery","Criteria","Patients","ORR,%", "95% CI"),
       xlab="Objective response rate,%", smlab="", fs.xlab=14, fs.study=12, fs.study.lables=12,fs.hetstat=12,
       ff.axis="bold.italic", ff.fixed="bold.italic", ff.random="bold.italic", ff.xlab="bold",
       weight.study="random", squaresize=0.7,
       col.square="navy",col.diamond="maroon", col.diamond.lines="maroon",
       pooled.totals=TRUE,
```

```
            print.Q=TRUE, print.pval.Q=TRUE,
            print.I2=TRUE, print.I2.ci = TRUE,
            print.tau2=TRUE, print.tau2.ci = TRUE,
            print.tau = TRUE, print.tau.ci=TRUE,
            calcwidth.pooled = TRUE, calcwidth.hetstat = TRUE,
            just = "center", col.by="grey", digits=1,
            digits.tau2=2, colgap.forest.left = "0.5inch")

funnel(pes.logit,atransf=transf.iarcsin,yaxis="sei", xlab="Proportion",digits=4,
            level=c(90, 95, 99), shade=c("white", "blue4", "lightblue3"), legend=TRUE)

metabias(pes.forest, method.bias = "peters", k.min=3)
```
